# Intelligence, educational attainment, and brain structure in those at familial high‐risk for schizophrenia or bipolar disorder

**DOI:** 10.1002/hbm.25206

**Published:** 2020-10-07

**Authors:** Sonja M. C. de Zwarte, Rachel M. Brouwer, Ingrid Agartz, Martin Alda, Silvia Alonso‐Lana, Carrie E. Bearden, Alessandro Bertolino, Aurora Bonvino, Elvira Bramon, Elizabeth E. L. Buimer, Wiepke Cahn, Erick J. Canales‐Rodríguez, Dara M. Cannon, Tyrone D. Cannon, Xavier Caseras, Josefina Castro‐Fornieles, Qiang Chen, Yoonho Chung, Elena De la Serna, Caterina del Mar Bonnin, Caroline Demro, Annabella Di Giorgio, Gaelle E. Doucet, Mehmet Cagdas Eker, Susanne Erk, Mar Fatjó‐Vilas, Scott C. Fears, Sonya F. Foley, Sophia Frangou, Janice M. Fullerton, David C. Glahn, Vina M. Goghari, Jose M. Goikolea, Aaron L. Goldman, Ali Saffet Gonul, Oliver Gruber, Tomas Hajek, Emma L. Hawkins, Andreas Heinz, Ceren Hidiroglu Ongun, Manon H. J. Hillegers, Josselin Houenou, Hilleke E. Hulshoff Pol, Christina M. Hultman, Martin Ingvar, Viktoria Johansson, Erik G. Jönsson, Fergus Kane, Matthew J. Kempton, Marinka M. G. Koenis, Miloslav Kopecek, Bernd Krämer, Stephen M. Lawrie, Rhoshel K. Lenroot, Machteld Marcelis, Venkata S. Mattay, Colm McDonald, Andreas Meyer‐Lindenberg, Stijn Michielse, Philip B. Mitchell, Dolores Moreno, Robin M. Murray, Benson Mwangi, Leila Nabulsi, Jason Newport, Cheryl A. Olman, Jim van Os, Bronwyn J. Overs, Aysegul Ozerdem, Giulio Pergola, Marco M. Picchioni, Camille Piguet, Edith Pomarol‐Clotet, Joaquim Radua, Ian S. Ramsay, Anja Richter, Gloria Roberts, Raymond Salvador, Aybala Saricicek Aydogan, Salvador Sarró, Peter R. Schofield, Esma M. Simsek, Fatma Simsek, Jair C. Soares, Scott R. Sponheim, Gisela Sugranyes, Timothea Toulopoulou, Giulia Tronchin, Eduard Vieta, Henrik Walter, Daniel R. Weinberger, Heather C. Whalley, Mon‐Ju Wu, Nefize Yalin, Ole A. Andreassen, Christopher R. K. Ching, Sophia I. Thomopoulos, Theo G. M. van Erp, Neda Jahanshad, Paul M. Thompson, René S. Kahn, Neeltje E. M. van Haren

**Affiliations:** ^1^ Department of Psychiatry University Medical Center Utrecht Brain Center, University Medical Center Utrecht, Utrecht University Utrecht Netherlands; ^2^ Norwegian Centre for Mental Disorders Research (NORMENT), Institute of Clinical Medicine, University of Oslo Oslo Norway; ^3^ Centre for Psychiatry Research, Department of Clinical Neuroscience Karolinska Institutet & Stockholm Health Care Services, Stockholm Region Stockholm Sweden; ^4^ Department of Psychiatry Diakonhjemmet Hospital Oslo Norway; ^5^ Department of Psychiatry Dalhousie University Halifax Nova Scotia Canada; ^6^ National Institute of Mental Health Klecany Czech Republic; ^7^ FIDMAG Germanes Hospitalàries Research Foundation Barcelona Spain; ^8^ CIBERSAM (Centro de Investigación Biomédica en Red de Salud Mental) Madrid Spain; ^9^ Semel Institute for Neuroscience and Human Behavior, University of California California Los Angeles USA; ^10^ Department of Psychology University of California California Los Angeles USA; ^11^ Department of Basic Medical Science, Neuroscience and Sense Organs University of Bari 'Aldo Moro' Bari Italy; ^12^ Division of Psychiatry, Neuroscience in Mental Health Research Department University College London London UK; ^13^ Centre for Neuroimaging & Cognitive Genomics (NICOG), Clinical Neuroimaging Laboratory, NCBES Galway Neuroscience Centre, College of Medicine Nursing and Health Sciences, National University of Ireland Galway Galway Ireland; ^14^ Department of Psychology Yale University New Haven Connecticut USA; ^15^ Department of Psychiatry Yale University School of Medicine New Haven Connecticut USA; ^16^ MRC Centre for Neuropsychiatric Genetics and Genomics Cardiff University Cardiff UK; ^17^ Department of Child and Adolescent Psychiatry and Psychology, 2017SGR881 Institute of Neuroscience, Hospital Clínic of Barcelona Barcelona Spain; ^18^ Institut d'Investigacions Biomèdiques August Pi i Sunyer (IDIBAPS) Barcelona Spain; ^19^ University of Barcelona Barcelona Spain; ^20^ Lieber Institute for Brain Development, Johns Hopkins Medical Campus Baltimore Maryland USA; ^21^ Bipolar and Depressive Disorders Unit Hospital Clinic, University of Barcelona Barcelona Spain; ^22^ Department of Psychiatry and Behavioral Sciences University of Minnesota Minneapolis Minnesota USA; ^23^ IRCCS Casa Sollievo della Sofferenza San Giovanni Rotondo Italy; ^24^ Department of Psychiatry Icahn School of Medicine at Mount Sinai New York New York USA; ^25^ Boys Town National Research Hospital Omaha NE USA; ^26^ SoCAT LAB, Department of Psychiatry School of Medicine, Ege University Izmir Turkey; ^27^ Research Division of Mind and Brain, Department of Psychiatry and Psychotherapy Charité Universitätsmedizin Berlin, Freie Universität Berlin, Humboldt‐Universität zu Berlin, and Berlin Institute of Health Berlin Germany; ^28^ Department of Psychiatry and Biobehavioral Science University of California Los Angeles California USA; ^29^ Center for Neurobehavioral Genetics University of California Los Angeles California USA; ^30^ Cardiff University Brain Research Imaging Centre, Cardiff University Cardiff UK; ^31^ Neuroscience Research Australia Sydney Australia; ^32^ School of Medical Sciences, University of New South Wales Sydney Australia; ^33^ Olin Neuropsychiatry Research Center, Institute of Living, Hartford Hospital Hartford Connecticut USA; ^34^ Tommy Fuss Center for Neuropsychiatric Disease Research Boston Children's Hospital Boston Massachusetts USA; ^35^ Harvard Medical School Boston Massachusetts USA; ^36^ Department of Psychology and Graduate Department of Psychological Clinical Science University of Toronto Toronto Ontario Canada; ^37^ Department of Psychiatry and Behavioral Sciences Mercer University School of Medicine Macon Georgia USA; ^38^ Section for Experimental Psychopathology and Neuroimaging, Department of General Psychiatry University of Heidelberg Heidelberg Germany; ^39^ Division of Psychiatry Royal Edinburgh Hospital, University of Edinburgh Edinburgh UK; ^40^ Department of Psychology, Faculty of Arts Dokuz Eylül University İzmir Turkey; ^41^ Department of Child and Adolescent Psychiatry/Psychology Erasmus University Medical Center‐Sophia Children's Hospital Rotterdam Netherlands; ^42^ APHP, Mondor University Hospitals Créteil France; ^43^ INSERM U955 Team 15 "Translational Psychiatry" Créteil France; ^44^ NeuroSpin neuroimaging platform, Psychiatry Team, UNIACT Lab, CEA Saclay Gif‐Sur‐Yvette France; ^45^ Department of Medical Epidemiology and Biostatistics Karolinska Institutet Stockholm Sweden; ^46^ Section for Neuroscience, Department of Clinical Neuroscience Karolinska Institutet Stockholm Sweden; ^47^ Department of Neuroradiology Karolinska University Hospital Stockholm Sweden; ^48^ Psychosis Studies, Institute of Psychiatry, Psychology and Neuroscience (IoPPN), King's College London London UK; ^49^ Department of Psychiatry, Third Faculty of Medicine Charles University Prague Czech Republic; ^50^ School of Psychiatry, University of New South Wales Sydney Australia; ^51^ Department of Psychiatry and Behavioral Sciences University of New Mexico Albuquerque New Mexico USA; ^52^ Department of Psychiatry and Neuropsychology School for Mental Health and Neuroscience, Maastricht University Medical Centre, Maastricht University Maastricht Netherlands; ^53^ Departments of Neurology and Radiology Johns Hopkins University School of Medicine Baltimore Maryland USA; ^54^ Department of Psychiatry and Psychotherapy Central Institute of Mental Health, Medical Faculty Mannheim, University of Heidelberg Mannheim Germany; ^55^ Child and Adolescent Psychiatry Department Hospital General Universitario Gregorio Marañón (IiSGM), School of Medicine, Universidad Complutense Madrid Spain; ^56^ Department of Psychiatry and Behavioral Sciences The University of Texas Health Science Center at Houston Houston Texas USA; ^57^ Department of Psychology and Center for Magnetic Resonance Research University of Minnesota Minneapolis Minnesota USA; ^58^ Department of Psychiatry, Faculty of Medicine Dokuz Eylül University Izmir Turkey; ^59^ Department of Neurosciences Health Sciences Institute, Dokuz Eylül University Izmir Turkey; ^60^ Department of Psychiatry and Psychology Mayo Clinic Rochester Minnesota USA; ^61^ Department of Forensic and Neurodevelopmental Science Institute of Psychiatry, Psychology and Neuroscience, King's College London London UK; ^62^ Department of Psychiatry, Faculty of Medicine University of Geneva Geneva Switzerland; ^63^ School of Medicine, Universitat Internacional de Catalunya Barcelona Spain; ^64^ Early Psychosis: Interventions and Clinical‐detection (EPIC) lab, Department of Psychosis Studies Institute of Psychiatry, Psychology & Neuroscience, King's College London London UK; ^65^ Department of Psychiatry, Faculty of Medicine Izmir Katip Çelebi University Izmir Turkey; ^66^ Institute of Psychiatry, Psychology and Neuroscience, King's College London London UK; ^67^ Cigli State Hospital Department of Psychiatry Izmir Turkey; ^68^ Minneapolis VA Health Care System Minneapolis Minnesota USA; ^69^ Department of Psychology Bilkent University Ankara Turkey; ^70^ Department of Basic and Clinical Neuroscience Institute of Psychiatry, Psychology and Neuroscience, King's College London London UK; ^71^ Centre for Affective Disorders, Department of Psychological Medicine Institute of Psychiatry, Psychology and Neuroscience, King's College London London UK; ^72^ Division of Mental Health and Addiction Oslo University Hospital Oslo Norway; ^73^ Imaging Genetics Center, Mark and Mary Stevens Neuroimaging and Informatics Institute, Keck School of Medicine, University of Southern California Marina del Rey California USA; ^74^ Clinical Translational Neuroscience Laboratory, Department of Psychiatry and Human Behavior University of California Irvine Irvine California USA; ^75^ Center for the Neurobiology of Learning and Memory University of California Irvine Irvine California USA

**Keywords:** bipolar disorder, education, intelligence, neuroimaging, relatives, schizophrenia

## Abstract

First‐degree relatives of patients diagnosed with schizophrenia (SZ‐FDRs) show similar patterns of brain abnormalities and cognitive alterations to patients, albeit with smaller effect sizes. First‐degree relatives of patients diagnosed with bipolar disorder (BD‐FDRs) show divergent patterns; on average, intracranial volume is larger compared to controls, and findings on cognitive alterations in BD‐FDRs are inconsistent. Here, we performed a meta‐analysis of global and regional brain measures (cortical and subcortical), current IQ, and educational attainment in 5,795 individuals (1,103 SZ‐FDRs, 867 BD‐FDRs, 2,190 controls, 942 schizophrenia patients, 693 bipolar patients) from 36 schizophrenia and/or bipolar disorder family cohorts, with standardized methods. Compared to controls, SZ‐FDRs showed a pattern of widespread thinner cortex, while BD‐FDRs had widespread larger cortical surface area. IQ was lower in SZ‐FDRs (*d* = −0.42, *p* = 3 × 10^−5^), with weak evidence of IQ reductions among BD‐FDRs (*d* = −0.23, *p* = .045). Both relative groups had similar educational attainment compared to controls. When adjusting for IQ or educational attainment, the group‐effects on brain measures changed, albeit modestly. Changes were in the expected direction, with less pronounced brain abnormalities in SZ‐FDRs and more pronounced effects in BD‐FDRs. To conclude, SZ‐FDRs and BD‐FDRs show a differential pattern of structural brain abnormalities. In contrast, both had lower IQ scores and similar school achievements compared to controls. Given that brain differences between SZ‐FDRs and BD‐FDRs remain after adjusting for IQ or educational attainment, we suggest that differential brain developmental processes underlying predisposition for schizophrenia or bipolar disorder are likely independent of general cognitive impairment.

## Graphical abstract

Family members of patients with schizophrenia (SZ‐FDRs) and bipolar disorder (BD‐FDRs) can provide insight into the effect of familial risk of these disorders on the brain and cognition. Here, we performed a meta‐analysis of global and regional brain measures, IQ, and educational attainment in 5,795 individuals. Both SZ‐FDRs and BD‐FDRs showed structural brain abnormalities, but in different regions and directions. In contrast, both had lower IQ scores yet similar school achievements compared to controls. Given that brain differences between SZ‐FDRs and BD‐FDRs remain after adjusting for IQ or educational attainment, we suggest that differential brain developmental processes underlying predisposition for schizophrenia or bipolar disorder are likely independent of general cognitive impairment.

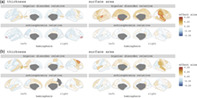

## 1 INTRODUCTION

Schizophrenia and bipolar disorder are highly heritable disorders with a shared genetic architecture (Anttila et al., 2018; Lee et al., 2013; Lichtenstein et al., 2009). Both patient groups are characterized by overlapping patterns of structural brain abnormalities (Arnone et al., 2009; Ellison‐Wright & Bullmore, 2010; Haijma et al., 2013; Hibar et al., 2016; Hibar et al., 2018; Ivleva et al., 2017; McDonald et al., 2004; Okada et al., 2016; van Erp et al., 2016, 2018). In contrast, our recent ENIGMA–Relatives meta‐analysis showed that their family members—who share the risk for the disorder but generally are not confounded by medication use or other illness related factors—show divergent patterns of global brain measures (de Zwarte, Brouwer, Agartz, et al., 2019). That study found that first‐degree relatives of patients diagnosed with bipolar disorder (BD‐FDRs) had a larger intracranial volume (ICV) which was not present in first‐degree relatives of patients diagnosed with schizophrenia (SZ‐FDRs). When we adjusted for ICV, no differences were found between BD‐FDRs and controls but SZ‐FDRs still showed significantly smaller brain volumes, diminished cortical thickness and larger ventricle volume compared to controls. These findings suggest that individuals at familial risk for either bipolar disorder or schizophrenia may show disease‐specific deviations during early brain development.

Differential neurodevelopmental trajectories in schizophrenia and bipolar disorder have also been linked to intelligence quotient (IQ) development and school performance (Parellada, Gomez‐Vallejo, Burdeus, & Arango, 2017). Schizophrenia has been associated with poorer cognitive performance, as well as decreases in cognitive performance over time, years before onset (Agnew‐Blais & Seidman, 2013; Dickson, Laurens, Cullen, & Hodgins, 2012; Hochberger et al., 2018; Kendler, Ohlsson, Sundquist, & Sundquist, 2015; Khandaker, Barnett, White, & Jones, 2011; Reichenberg et al., 2005; Woodberry, Giuliano, & Seidman, 2008), while premorbid IQ or educational attainment are often not affected or are even higher in individuals who later develop bipolar disorder (MacCabe et al., 2010; Smith et al., 2015; Tiihonen et al., 2005; Zammit et al., 2004).

Both IQ and educational attainment are highly heritable (Devlin, Daniels, & Roeder, 1997; Heath et al., 1985; Tambs, Sundet, Magnus, & Berg, 1989). Consequently, similar patterns of cognitive performance and educational attainment are often found among relatives. Indeed, cognitive alterations have been reported in SZ‐FDRs compared to controls (Hughes et al., 2005; Kremen, Faraone, Seidman, Pepple, & Tsuang, 1998; McIntosh, Harrison, Forrester, Lawrie, & Johnstone, 2005; Niendam et al., 2003; Sitskoorn, Aleman, Ebisch, Appels, & Kahn, 2004; Van Haren, Van Dam, & Stellato, 2019; Vreeker et al., 2016) and in BD‐FDRs compared to controls (Vonk et al., 2012; Vreeker et al., 2016). Vreeker et al. (2016) showed, in a direct comparison, a discrepancy between IQ and educational attainment in SZ‐FDRs and BD‐FDRs: both groups showed lower IQ but similar educational attainment compared to controls. These findings suggest that, despite the high genetic and phenotypic overlap between intelligence and educational attainment in the general population (Sniekers et al., 2017; Strenze, 2007), it is important to differentiate between these two measures when investigating individuals at familial risk for mental illness.

Intelligence has consistently been associated with brain structure in the general population (McDaniel, 2005). Our recent schizophrenia family studies reported that IQ is intertwined with most of the brain abnormalities in SZ‐FDRs (de Zwarte, Brouwer, Tsouli, et al., 2019; van Haren et al., 2020). Altered brain structure and cognitive deficits observed in schizophrenia patients could be a direct consequence of the association observed in the general population or alternatively, both could be caused by the disease through independent mechanisms. IQ and brain structure are both considered indirect measures for (early) neurodevelopment. Indeed, both brain structure and cognitive deficits in schizophrenia relatives are apparent in children and adolescents at high familial risk (van Haren et al., 2020), suggesting that individuals at familial risk for schizophrenia show altered neurodevelopment already early in life. This would suggest that genetic risk for the disease influences both cognition and the brain and we cannot study one without the other. However, in BD‐FDRs, it remains unknown how IQ and risk for bipolar disorder interact with the brain. In particular, the relationship between IQ and the familial predisposition for a larger ICV in BD‐FDRs is unclear. Hence, it is of interest to see how brain anomalies and intelligence differences are related to each other in those at familial risk for schizophrenia and bipolar disorder.

Here, through the Enhancing Neuro Imaging Genetics Through Meta Analysis (ENIGMA)‐Relatives Working Group, we performed meta‐analyses of magnetic resonance imaging data sets consisting of SZ‐FDRs and/or BD‐FDRs, probands, and matched control participants. There were three main aims. First, we extended our findings of group differences in global brain measures between relatives and controls (and patients) for both disorders (de Zwarte, Brouwer, Agartz, et al., 2019) by adding local cortical measures. This allowed us to investigate whether the findings were limited to specific (functional) brain regions, or can be attributed to a more global mechanism. Previous ENIGMA meta‐analyses have shown that patients with schizophrenia have widespread attenuation of cortical thickness and surface area (with largest effects in frontal and temporal lobe regions), with evidence for regional specificity only in the thickness findings (van Erp et al., 2018). In contrast, patients with bipolar disorder have shown thinner cortex in frontal, temporal and parietal regions, but no differences in surface area, compared to controls (Hibar et al., 2018). Based on the patient findings and our previous ENIGMA‐Relatives findings for global brain measures—showing globally thinner cortex in SZ‐FDRs and larger surface area in BD‐FDRs—we expected to find subtle regional differences in these measures in the relatives. In particular, we predicted locally thinner cortex in SZ‐FDRs with a similar pattern to previous observations in patients but with smaller effect sizes (van Erp et al., 2018). Based on the larger ICV and global surface area reported in our previous study, locally larger surface area in BD‐FDRs was expected in contrast to previous bipolar patient findings (Hibar et al., 2018). Second, in cohorts that had information on current IQ and/or educational attainment (the latter is defined as years of education completed), we meta‐analyzed the group effects of IQ and educational attainment between relatives and controls (and patients) for both disorders. We hypothesized that both SZ‐FDRs and BD‐FDRs would have, on average, lower current IQ than controls. Educational attainment findings in relatives have been inconsistent, with findings of both lower educational attainment and no detectable differences between relatives and controls; therefore, we expected subtle but significant differences between both SZ‐FDRs and BD‐FDRs and controls. Third, we investigated the influence of IQ and educational attainment on global and local brain differences between relatives and controls. We hypothesized that IQ will account for most of the brain abnormalities found in SZ‐FDRs, while a lower IQ most likely would not explain our previously reported larger ICV in BD‐FDRs because of the well‐ established positive relationship between overall head size and IQ (McDaniel, 2005). The moderating effect of educational attainment on brain abnormalities is expected to be less pronounced than that of IQ, as we are only expecting modest group differences in educational attainment between relatives and controls.

## 2 MATERIALS AND METHODS

### 2.1 Study samples

This study included 5,795 participants from 36 family cohorts (age range 6–72 years). In total, 1,103 SZ‐FDRs (42 monozygotic co‐twins, 50 dizygotic co‐twins, 171 offspring, 728 siblings, 112 parents), 867 BD‐FDRs (32 monozygotic co‐twins, 33 dizygotic co‐twins, 453 offspring, 331 siblings, 18 parents), 942 patients with schizophrenia, 693 patients with bipolar disorder and 2,190 controls were included (Tables 1 and 2). All family cohorts included their own control participants. Controls did not have a family history of schizophrenia or bipolar disorder. SZ‐FDRs or BD‐FDRs were defined by having a first‐degree family member with schizophrenia or bipolar disorder, respectively, and not having experienced (hypo)mania and/or psychosis themselves. Demographic characteristics for each cohort and their inclusion criteria are summarized in Tables 1 and 2 and Table S1. The cohorts in the current meta‐analysis overlap largely, but not completely with those in our previous meta‐analysis (de Zwarte, Brouwer, Agartz, et al., 2019). All study centers obtained approval from their respective ethics committee for research, following the Declaration of Helsinki. Informed consent was obtained from all participants and/or parents, in the case of minors.

**TABLE 1:** Sample demographics bipolar disorder family cohorts

	Total									IQ scores						Educational attainment					
	Controls			Patients			Relatives			Controls		Patients		Relatives		Controls		Patients		Relatives	
Sample	*N*	M/F	Age	*N*	M/F	Age	*N*	M/F	Age	*N*	IQ	*N*	IQ	*N*	IQ	*N*	EA	*N*	EA	*N*	EA
BPO‐FLB	7	3/4	12.9 (1.3)	9	5/4	13.3 (2.6)	22	10/12	10.0 (3.5)	7	91.0 (10.2)	5	91.2 (16.1)	7	95.4 (17.3)	—		—		—	
Cardiff	79	28/51	39.8 (8.7)	120	42/78	41.9 (8.1)	33	13/20	45.9 (6.9)	—		—		—		—		—		—	
CliNG‐BD^a^	19	6/13	30.9 (9.6)	—			19	6/13	31.9 (5.0)	—		—		—		12	14.9 (3.3)	—		10	15.2 (3.2)
DEU	27	11/16	32.9 (8.8)	27	10/17	36.3 (9.5)	23	11/12	31.3 (8.9)	—		—		—		21	13.1 (4.1)	24	12.9 (2.9)	14	11.6 (3.1)
EGEU	33	13/20	33.6 (7.8)	27	16/11	36.7 (7.8)	27	10/17	34.5 (9.5)	—		—		—		28	11.6 (3.8)	26	10.8 (4.2)	23	10.8 (4.4)
ENBD‐UT	36	13/23	34.8 (11.7)	72	23/49	36.9 (12.4)	52	10/42	44.3 (13.6)	27	101.0 (14.5)	40	97.0 (12.0)	19	99.2 (14.4)	26	15.2 (3.0)	55	14.7 (2.3)	46	15.1 (2.3)
FIDMAG‐Clinic	61	12/49	41.1 (10.1)	18	3/15	42.6 (8.8)	18	5/13	45.1 (10.0)	61	112.9 (13.6)	14	101.9 (13.1)	16	105.8 (16.7)	—		—		—	
Geneva	19	10/9	20.1 (2.7)	—			18	9/9	19.4 (3.1)	—		—		—		—		—		—	
IDIBAPS^a^	53	21/32	12.3 (3.6)	—			61	31/30	12.4 (3.4)	53	106.1 (12.4)	—		61	107.0 (13.0)	—		—		—	
IoP‐BD	39	9/30	35.4 (11.2)	34	15/19	40.6 (13.1)	17	4/13	43.1 (14.6)	—		—		—		31	15.2 (2.6)	26	15.4 (3.2)	14	16.4 (2.6)
MFS‐BD^a^	54	25/29	40.2 (15.3)	38	15/23	41.0 (11.7)	41	17/24	49.3 (9.6)	39	110.8 (16.1)	31	97.4 (11.7)	34	100.0 (10.3)	35	14.1 (3.9)	35	13.9 (3.3)	31	14.6 (4.0)
MooDS‐BD^a^	63	25/38	30.3 (9.5)	—			63	25/38	30.4 (9.4)	62	99.4 (5.5)	—		62	101.5 (5.8)	33	15.4 (2.4)	—		34	17.2 (2.8)
MSSM	52	25/27	35.2 (13.0)	41	21/20	44.3 (11.9)	50	26/24	33.8 (8.3)	—		—		—		—		—		—	
Olin	68	25/43	32.2 (11.7)	108	34/74	34.5 (12.3)	78	30/48	32.0 (13.0)	54	107.0 (15.0)	95	102.9 (15.6)	68	105.6 (15.1)	40	15.2 (2.4)	74	14.6 (2.2)	40	14.6 (2.2)
ORBIS‐I	32	12/20	20.7 (3.3)	6	0/6	22.9 (4.0)	39	13/26	19.8 (3.2)	—		—		—		—		—		—	
ORBIS‐II	18	7/11	23.0 (3.5)	8	3/5	24.0 (5.0)	26	10/16	19.9 (4.0)	—		—		—		—		—		—	
PENS‐BD^a^	16	6/10	45.9 (10.1)	20	14/6	46.9 (10.4)	9	5/4	40.4 (6.3)	16	115.7 (13.8)	20	103.7 (15.8)	9	101.3 (18.0)	16	16.0 (1.3)	19	14.8 (2.7)	9	15.0 (1.6)
PHCP‐BD^a^	38	21/17	38.4 (13.7)	29	7/22	32.2 (11.6)	7	2/5	51.0 (6.1)	38	106.3 (11.8)	29	101.8 (8.8)	7	100.6 (9.1)	29	16.0 (2.5)	18	14.8 (1.8)	7	15.4 (1.5)
STAR‐BD^a^	83	39/44	49.0 (10.4)	25	7/18	45.8 (10.1)	21	6/15	47.9 (11.3)	—		—		—		81	11.9 (2.9)	25	12.9 (3.6)	21	11.5 (2.5)
SydneyBipolarGroup	117	54/63	22.2 (3.9)	59	17/42	25.1 (3.6)	150	65/85	19.9 (5.4)	116	117.6 (10.3)	57	116.2 (12.3)	147	114.5 (10.6)	24	17.1 (3.2)	32	16.4 (2.3)	30	15.9 (2.2)
UMCU‐BD twins^a^	110	40/70	39.3 (9.2)	52	13/39	39.6 (9.7)	27	9/18	41.7 (9.3)	48	98.0 (13.5)	22	92.4 (13.2)	14	95.4 (14.1)	108	13.4 (2.7)	47	12.7 (2.6)	26	12.1 (2.6)
UMCU‐DBSOS^a^	40	21/19	12.7 (2.1)	—			66	37/29	14.7 (2.7)	40	117.1 (13.0)	—		63	106.7 (18.3)	—		—		—	

^a^ Overlapping controls with schizophrenia sample from the same site, that is, with CliNG‐SZ (*n* = 10), IDIBAPS (*n* = 53), MFS‐SZ (*n* = 54), MooDS‐SZ (*n* = 36), PENS‐SZ (*n* = 16), PHCP‐SZ (*n* = 38), STAR‐SZ (*n* = 73), UMCU‐UTWINS (*n* = 19), UMCU‐DBSOS (*n* = 40).

**TABLE 2:** Sample demographics schizophrenia family cohorts

	Total									IQ scores						Educational attainment					
	Controls			Patients			Relatives			Controls		Patients		Relatives		Controls		Patients		Relatives	
Sample	*N*	M/F	Age	*N*	M/F	Age	*N*	M/F	Age	*N*	IQ	*N*	IQ	*N*	IQ	*N*	EA	*N*	EA	*N*	EA
C‐SFS	23	11/12	40.2 (11.1)	25	13/12	40.8 (10.8)	23	8/15	42.1 (11.9)	—		—		—		20	15.2 (2.4)	23	14.2 (3.1)	19	16.1 (2.7)
CliNG‐SZ^a^	20	11/9	35.7 (12.2)	—			20	11/9	36.1 (6.4)	—		—		—		14	15.1 (2.1)	—		14	14.0 (2.6)
EHRS	89	44/45	21.0 (2.5)	31	19/12	21.8 (3.7)	90	44/46	21.2 (3.1)	82	101.9 (12.9)	22	87.9 (14.5)	90	97.6 (13.5)	—		—		—	
HUBIN	102	69/33	41.9 (8.9)	104	78/26	41.3 (7.7)	33	23/10	39.4 (7.8)	69	102.0 (16.5)	73	89.1 (20.4)	19	106.6 (12.4)	90	14.3 (3.0)	93	12.5 (2.7)	30	12.9 (2.3)
IDIBAPS^a^	53	21/32	12.3 (3.6)	—			37	21/16	11.0 (3.3)	53	106.1 (12.4)	—		37	97.6 (14.2)	—		—		—	
IoP‐SZ	67	35/32	40.8 (12.2)	54	39/15	34.8 (10.8)	18	8/10	33.0 (12.4)	41	119.6 (13.9)	37	91.5 (15.8)	12	102.2 (12.6)	57	14.1 (2.4)	41	13.3 (3.1)	14	13.5 (2.9)
LIBD	361	162/199	32.5 (9.9)	211	161/50	35.2 (10.2)	240	99/141	36.2 (9.6)	361	109.6 (9.2)	211	95.4 (11.6)	240	107.3 (10.8)	259	17.5 (2.7)	165	14.8 (2.4)	201	16.3 (2.4)
Maastricht‐GROUP	87	33/54	30.8 (10.8)	88	59/29	28.2 (7.0)	96	50/46	29.5 (8.7)	87	111.3 (15.0)	87	96.7 (14.3)	96	108.9 (16.2)	—		—		—	
MFS‐SZ^a^	54	25/29	40.2 (15.3)	42	31/11	36.4 (9.8)	56	21/35	49.4 (8.4)	35	107.8 (14.1)	39	106.4 (16.1)	35	107.9 (16.8)	35	14.1 (3.9)	39	13.9 (3.2)	41	14.1 (3.0)
MooDS‐SZ^a^	65	26/39	30.6 (10.1)	—			63	24/39	30.6 (8.2)	63	100.0 (5.0)	—		61	97.5 (12.3)	37	15.1 (2.3)	—		35	16.1 (2.5)
PENS‐SZ^a^	16	6/10	45.9 (10.1)	20	13/7	47.4 (9.5)	11	4/7	48.3 (8.9)	16	115.7 (13.8)	20	102.9 (15.3)	11	105.0 (14.8)	16	16.0 (1.3)	19	12.7 (1.6)	11	14.5 (1.8)
PHCP‐SZ^a^	38	21/17	38.4 (13.7)	41	30/11	42.2 (11.6)	13	4/9	45.4 (11.4)	38	106.3 (11.8)	41	93.3 (11.7)	13	99.5 (10.5)	29	16.0 (2.5)	38	13.8 (2.3)	12	15.8 (3.0)
STAR‐SZ^a^	73	33/40	49.0 (10.4)	31	18/13	49.7 (8.9)	29	17/12	49.8 (9.6)	—		—		—		73	12.0 (3.0)	31	12.9 (3.4)	28	12.0 (3.8)
UMCU‐DBSOS^a^	40	21/19	12.7 (2.1)	—			40	12/28	13.7 (3.0)	40	117.1 (13.0)	—		40	100.6 (19.2)	—		—		—	
UMCU‐GROUP	167	83/84	27.7 (8.2)	162	130/32	27.0 (5.8)	201	95/106	27.7 (7.1)	164	111.9 (14.8)	153	93.5 (15.5)	199	101.4 (14.3)	83	14.0 (2.1)	83	11.2 (3.0)	119	13.5 (2.7)
UMCU‐Parents	41	14/27	52.8 (4.6)	—			44	13/31	52.9 (4.3)	41	119.0 (13.1)	—		44	116.9 (14.7)	41	12.5 (3.1)	—		43	12.1 (3.8)
UMCU‐UTWINS^a^	184	84/100	31.8 (13.0)	56	33/23	35.6 (10.6)	45	29/16	37.0 (11.9)	168	106.0 (13.3)	45	96.7 (15.0)	38	107.2 (15.1)	94	13.9 (2.4)	39	11.4 (3.4)	34	13.1 (2.9)
UNIBA	78	52/26	31.4 (8.6)	77	58/19	33.9 (8.2)	44	23/21	33.8 (8.9)	64	108.1 (12.7)	60	74.5 (17.0)	33	94.6 (17.2)	22	15.7 (3.3)	45	11.4 (3.3)	13	13.0 (4.4)

^a^ Overlapping controls with bipolar sample from the same site, i.e. with CliNG‐BD (*n* = 10), IDIBAPS (*n* = 53), MFS‐BD (*n* = 54), MooDS‐BD (*n* = 36), PENS‐BD (*n* = 16), PHCP‐BD (*n* = 38), STAR‐BD (*n* = 73), UMCU‐BD twins (*n* = 19), UMCU‐DBSOS (*n* = 40).

### 2.2 Intelligence quotient

Twenty‐five family cohorts had either full scale IQ scores or estimated IQ scores available for most of their participants. In total, 4,095 participants with a measure of IQ were included; 968 SZ‐FDRs, 507 BD‐FDRs, 788 patients with schizophrenia, 313 patients with bipolar disorder and 1,549 controls (Tables 1 and 2; Table S2 for IQ test battery description).

### 2.3 Educational attainment

Educational attainment was measured as years of completed education. These data were available in 27 family cohorts. Subjects were included if they were at least 25 years old to avoid the bias of including participants still in school. In total, 3,056 participants were included; 614 SZ‐FDRs, 306 BD‐FDRs, 616 patients with schizophrenia, 381 patients with bipolar disorder and 1,139 controls (Tables 1 and 2; Table S3 for educational attainment description).

### 2.4 Image acquisition and processing

Structural T1‐weighted brain magnetic resonance imaging scans were acquired at each research center (Table S4). Cortical and subcortical reconstruction and volumetric segmentations were performed with the FreeSurfer pipeline (Table S4) (http://surfer.nmr.mgh.harvard.edu/fswiki/recon-all/; Fischl, 2012). The segmentations were quality checked according to the ENIGMA quality control protocol for subcortical volumes, cortical thickness and surface area (http://enigma.ini.usc.edu/protocols/imaging‐protocols/). Global brain measures, regional cortical thickness, and surface area measures and subcortical volumes were extracted from individual images (Fischl & Dale, 2000; Fischl, Sereno, & Dale, 1999).

### 2.5 Statistical meta‐analyses

All statistical analyses were performed using R (http://www.rproject.org). Linear mixed model analyses were performed within each cohort for bipolar disorder and schizophrenia separately, comparing relatives (per relative type) with controls and, if present, patients with controls, while taking family relatedness into account (http://CRAN.R-project.org/package=nlme; Pinheiro & Bates, 2000). Patients were analyzed as a sanity check as effects in patients are not the main focus of the study; for differences between patients and controls we refer to Supporting Information. Mean centered age, age squared, and sex were included as covariates. Brain measures were corrected for lithium use at time of scan in patients with bipolar disorder by adding a covariate (yes = 1/no = 0). All global brain measures and subcortical volume analyses were performed both with and without adjusting for ICV by including ICV as covariate. No interaction terms were modeled. All regional cortical thickness analyses were performed with and without correction for mean cortical thickness and all regional cortical surface areas with and without correction for total surface area to assess regional specificity. Analyses of multiscanner studies included binary dummy covariates for *n* − 1 scanners. Cohen's *d* effect sizes and 95% confidence intervals were calculated within each cohort separately and pooled per disorder for all relatives combined, and for patients as a group, using an inverse variance‐weighted random‐effects meta‐analysis. All random‐effects models were fitted using the restricted maximum likelihood method. False discovery rate (*q* < 0.05) thresholding across all global and subcortical phenotypes, and separately per regional phenotype, was used to control for multiple comparisons for the analyses between relatives and controls, and between patients and controls (Benjamini & Hochberg, 1995). Correlations between brain measures and IQ, brain measures and educational attainment, and between IQ and educational attainment were estimated by performing linear mixed model analyses in the overall sample and in the relative groups only, based on the gathered statistics of the local analyses. The resulting *t*‐statistics were converted to correlation *r* with R package “esc” (http://CRAN.R-project.org/package=esc). Analyses were generally performed locally by the research center that contributed the cohort, using code created within the ENIGMA‐Relatives Working Group (scripts available upon request). For some cohorts, data were sent to the main site for analysis.

## 3 RESULTS

### 3.1 Cortical thickness

**FIGURE 1:**
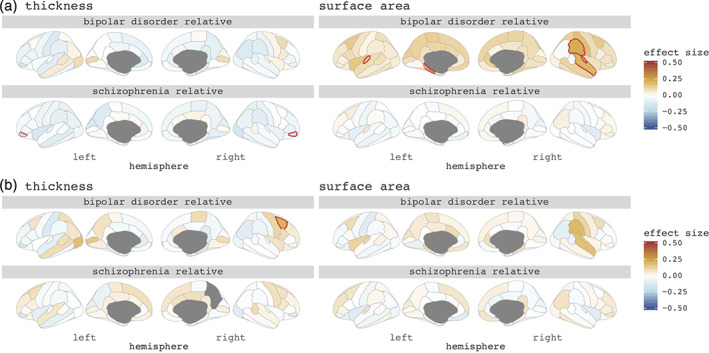
Cohen's *d* effect sizes comparing bipolar relatives and schizophrenia relatives to controls on (a) regional cortical thickness (left) and cortical surface area (right), (b) corrected for mean cortical thickness (left) and total surface area (right). Red lined regions survive false discovery rate correction for multiple testing (*q* < 0.05)

SZ‐FDRs had a thinner cortex in most cortical regions, compared to controls, with a thinner bilateral pars orbitalis surviving correction for multiple testing (left *d* = −0.17, right *d* = −0.16, *q* < 0.05 corrected; Figure 1a). There were no significant differences in regional cortical thickness in BD‐FDRs compared to controls.

To investigate whether findings were driven by a global effect we corrected for mean cortical thickness. None of the findings survived correction for multiple testing in SZ‐FDRs. BD‐FDRs had a significantly thicker right caudal middle frontal cortex (*d* = +0.21, *q* < 0.05 corrected) (Figure 1b). For all regional cortical thickness effect sizes, and for the patient findings, see Figure 1, and Figures S1 and S2.

### 3.2 Cortical surface area

Differences between SZ‐FDRs and controls were subtle and none were statistically significant. BD‐FDRs had larger cortical surface areas in many cortical areas compared to controls, with a significantly larger cortical surface area in the left transverse temporal, left parahippocampal, right superior temporal, right supramarginal and right transverse temporal regions surviving correction for multiple testing (*d*'s > +0.15, *q* < 0.05 corrected) (Figure 1a).

When controlling for total surface area to investigate regional specificity, none of the findings survived (Figure 1b). For all regional cortical surface area effect sizes, and for the patient findings see Figure 1, and Figures S3 and S4.

### 3.3 Intelligence quotient

**FIGURE 2:**
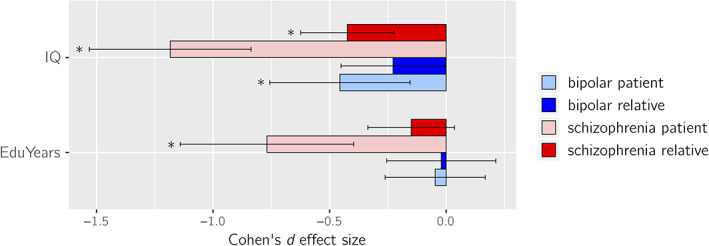
Cohen's *d* effect sizes comparing bipolar disorder patients (light blue), bipolar disorder relatives (blue), schizophrenia patients (pink), and schizophrenia relatives (red) to controls for intelligence quotient scores (IQ; top) and educational attainment (EduYears; bottom). The error bars depict the lower and upper 95% confidence intervals (CIs). *p < .001

**FIGURE 3:**
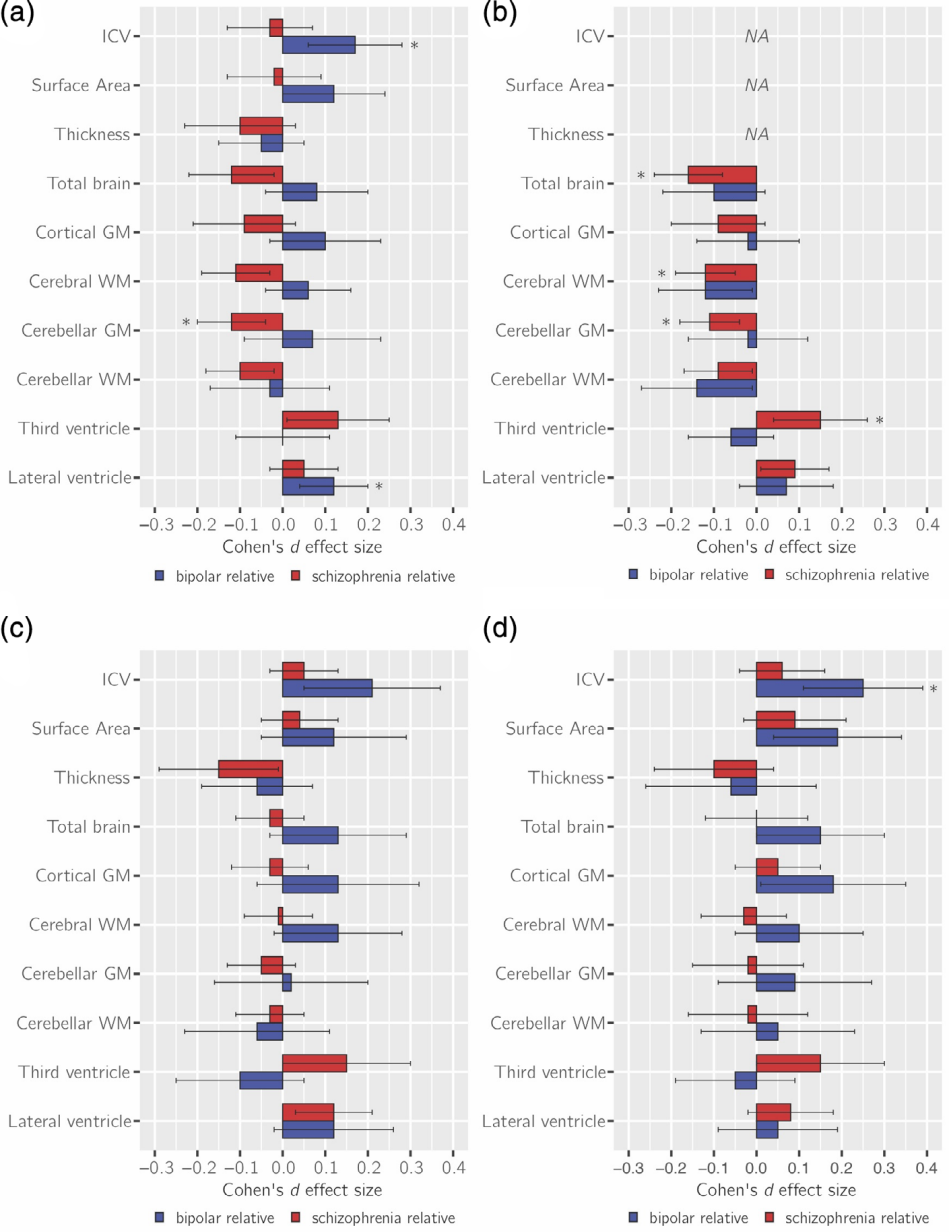
Cohen's *d* effect sizes comparing schizophrenia relatives (red), and bipolar disorder relatives (blue) to controls on (a) global brain measures, corrected for (b) intracranial volume (ICV), (c) intelligent quotient (IQ), (d) educational attainment. Analyses displayed in (a) and (b) have been presented in our previous study, but are repeated here, for completeness, albeit with slightly different cohorts (de Zwarte, Brouwer, Agartz, et al., 2019). Error bars depict the lower and upper 95% confidence intervals (CIs). *q < 0.05, corrected. GM, gray matter; NA, not corrected for ICV; WM, white matter

SZ‐FDRs had significantly lower IQ compared to controls with a medium effect size *d* = −0.42 (*p* = 3 × 10^−5^). BD‐FDRs showed mild IQ reductions compared to controls and of borderline significance with effect size *d* = −0.23 (*p* = .045; Figure 2). These findings translate to an average of 6.3 IQ points lower in SZ‐FDRs and 3.5 IQ points lower in BD‐FDRs compared to controls. In SZ‐FDRs, most effect sizes of the global brain measures were slightly smaller after controlling for IQ; none of them survived correction for multiple testing (Figure 3c, Table S5, Figures S5 and S6). After controlling for IQ, most effect sizes of the global brain measures were slightly larger in BD‐FDRs; however, after correction for multiple testing only larger caudate volume survived (*d* = +0.23; *q* < 0.05 corrected) (Figure 3c, Table S5, Figure S5 and S6). For all effect sizes and the effects in patients, see Table S5–S8, and Figure S5 and S6.

### 3.4 Educational attainment

Both SZ‐FDRs and BD‐FDRs did not differ from controls on years of education completed (Figure 2). After adjusting for educational attainment, the effect sizes in most global brain measures were slightly smaller in SZ‐FDRs (none of which survived correction for multiple testing), while the effect sizes of the global brain measures were slightly larger in BD‐FDRs, with a significantly larger ICV (*d* = +0.25, *q* < 0.05 corrected; Figure 3d, Table S5, Figure S5 and S6). For all effect sizes and the effects in patients, see Tables S5–S8, and Figure S5 and S6.

### 3.5 Correlations between IQ, educational attainment, and brain measures

The correlation between IQ and educational attainment in the total sample was *r* = .40 (*p* = 4 × 10^−22^). All correlations between IQ and global and subcortical brain measures were positive (ranging from *r* = .06 and *r* = .22 [*q* < 0.05 corrected], except for the third [*r* = −.04] and lateral ventricles [*r* = −.01]; Table S9; for the results in the SZ‐FDRs or BD‐FDRs subgroups see Table S10). A significant positive correlation was found between educational attainment and total brain, cortical gray matter, cerebellar gray and white matter, and hippocampal volume (*r* = .06–.08, *q* < 0.05 corrected; Table S9).

## 4 DISCUSSION

In previous work from the ENIGMA‐Relatives Working Group we showed that BD‐FDRs had a larger ICV which was not found in SZ‐FDRs; when we adjusted for ICV, no differences in global brain measures were found between BD‐FDRs and controls, while SZ‐FDRs had significantly smaller brain volumes, diminished cortical thickness, and larger ventricle volume compared to controls (de Zwarte, Brouwer, Agartz, et al., 2019). In this study, we extended the investigation to compare local cortical measures, IQ and educational attainment in SZ‐FDRs and BD‐FDRs with controls and investigated the effect of IQ and educational attainment on global and local brain measures in the relatives.

The main findings in the current study were that: (a) SZ‐FDRs had a thinner cortex in most cortical regions, compared to controls, with a thinner bilateral pars orbitalis surviving correction for multiple testing. However, these findings may reflect a global effect rather than regionally specific effect. In contrast, BD‐FDRs had a significantly thicker caudal middle frontal cortex when compared to controls that was only present when statistically controlling for global thickness and may thus reflect regionally specific sparing; (b) only BD‐FDRs (and not SZ‐FDRs) had larger cortical surface area in the temporal lobe, which was no longer present after statistically controlling for total surface area; (c) IQ was lower in both BD‐FDRs and SZ‐FDRs, while educational attainment did not differ between the relatives and controls; (d) there was a modest yet significant correlation between IQ and most brain measures in the full sample; however, statistically controlling for individual differences in IQ and educational attainment only minimally changed the group effects on the brain measures the expected direction, that is, effect sizes of brain measure differences between groups decreased for SZ‐FDRs and increased for BD‐FDRs after adjusting for IQ or educational attainment.

### 4.1 Cortical thickness and surface area in the relatives

SZ‐FDRs had a thinner cortex in most brain areas. This pattern of findings is comparable to that in the included patient sample (Figure S1) as well as in the much larger sample of patients diagnosed with schizophrenia in an earlier ENIGMA study (van Erp et al., 2018). However, effect sizes are lower in SZ‐FDRs. The most pronounced effect was observed in the bilateral *pars orbitalis*. This region has previously been associated with language function in those at familial risk for schizophrenia (Francis et al., 2012) and in individuals with nonclinical auditory verbal hallucinations (Van Lutterveld et al., 2014). When statistically controlling for mean cortical thickness this finding was no longer significant, suggesting that thinner cortex in SZ‐FDRs is a global effect. In contrast, the pattern in BD‐FDRs was diffuse with both thicker and thinner cortical regions, whereas bipolar patients showed globally thinner cortex (Figure S1), consistent with previous findings (Hibar et al., 2018). After correction for mean cortical thickness the right *caudal middle frontal cortex* was significantly thicker when compared to controls, suggesting regionally specific cortical thickness abnormalities in BD‐FDRs.

Regional cortical surface area findings showed, on the one hand, that where the patients with schizophrenia had overall smaller surface area (Figure S3; van Erp et al., 2018), the effects in SZ‐FDRs were even more subtle, and in both directions, compared to controls. On the other hand, BD‐FDRs had widespread larger regional cortical surface area when compared to controls, in accord with our previous findings of larger total surface area in BD‐FDRs (de Zwarte, Brouwer, Agartz, et al., 2019). While total surface area in patients with bipolar disorder was not significantly larger, we did see a pattern of mostly larger regional cortical surface area in the bipolar patients as well (Figure S3), with most prominent findings in the temporal lobe, in auditory regions associated with language (Crinion, Lambon‐Ralph, Warburton, Howard, & Wise, 2003; Saur et al., 2008). This finding was not reported in the large ENIGMA bipolar disorder meta‐analysis (Hibar et al., 2018); however, findings in that study were all corrected for ICV which most likely reduced the global surface area differences. In line with this, when we corrected for overall surface area, regional surface differences in BD‐FDRs was no longer significant, suggesting that the larger surface area in BD‐FRDs was a global effect.

Cortical thickness and surface area are highly heritable and largely influenced by independent genetic factors (Grasby et al., 2020; Strike et al., 2019). The latest cortical thickness and surface area genome‐wide association study (GWAS) showed that the effects of genetic variants associated with surface area are more likely to be prenatal, while cortical thickness effects are more likely postnatal (Grasby et al., 2020), supporting the radial unit hypothesis that cortical thickness and surface area originate from two distinct processes in early brain development (Rakic, 1988). That BD‐FDRs and SZ‐FDRs show different patterns of abnormal cortical thickness and surface area, strengthens the notion that genetic predisposition may underlie distinct neurodevelopmental trajectories for these disorders early in life.

### 4.2 Discrepancy between IQ and educational attainment

Given the high genetic (*r*_g_ = .7 [Sniekers et al., 2017]) and phenotypic (*r* = ~.5 [Strenze, 2007]) correlation between intelligence and educational attainment, educational attainment is often considered a proxy for IQ. In the current study, we found a phenotypic correlation of *r* = .4 between IQ and educational attainment. This implies that educational attainment is at most a weak proxy for IQ; it only explains 16% of the variance. We showed that IQ and educational attainment act differently in relatives, that is, lower IQ in SZ‐FDRs and BD‐FDRs than in controls, with larger alterations in SZ‐FDRs, but no differences in educational attainment between SZ‐FDRs and BD‐FDRs as compared to controls. These findings are in line with an earlier study—of which a subset of the participants is included in the present study—investigating IQ and educational attainment in SZ‐FDRs and BD‐FDRs (Vreeker et al., 2016), suggesting that even though relatives have a lower IQ on average, gross school performance and engagement is not necessarily affected. It has previously been shown that differentiating intelligence from educational performance is important, as other factors besides intelligence are predictive of educational performance (Chamorro‐Premuzic & Furnham, 2003; Deary, Strand, Smith, & Fernandes, 2007). In addition, completing a level of education gives little insight into the level of academic performance (e.g., grades). In fact, those measures are only partly correlated (Strenze, 2007). Perhaps, the modest cognitive alterations in the relatives cannot be picked up by a categorical measure such as educational attainment or the cognitive alterations must reach a certain threshold to lead to a lower level of school performance, which may be the case in patients with schizophrenia (who have the largest negative effect size for IQ and are significantly different from controls in educational attainment).

### 4.3 IQ, educational attainment and the brain

IQ and educational attainment both share genetic variance with ICV (*r*_g_ = .29 and *r*_g_ = .34, respectively (Okbay et al., 2016; Sniekers et al., 2017). Therefore, we speculated previously that the larger ICV reported in BD‐FDRs could potentially be confounded by higher cognitive functioning (de Zwarte, Brouwer, Agartz, et al., 2019; de Zwarte, Brouwer, Tsouli, et al., 2019). Here, we showed that in the total sample ICV and all global brain measures were significantly correlated with IQ, except the ventricles, while correlations between the brain measures and educational attainment were much smaller. Adding to that, IQ was significantly lower in the relatives while this was not the case for educational attainment. Based on these findings, we propose that IQ is a more informative measure than educational attainment to explain variation in brain measures or group differences in brain measures.

As mentioned, only small‐to‐modest effects of IQ in relation to brain abnormalities in those at familial risk were reported, but these were in the expected direction. In SZ‐FDRs, adjusting for IQ explained part of the effect of familial risk for schizophrenia in total brain, gray and white matter volumes (i.e., effect sizes decreased). This was previously shown in two twin studies (both included in this study; Bohlken, Brouwer, Mandl, Kahn, & Hulshoff Pol, 2016; Toulopoulou et al., 2015) and a study that included a subset of the present participants using a mega‐analysis (de Zwarte, Brouwer, Tsouli, et al., 2019). Interestingly, adjusting for IQ resulted in an even larger ICV difference in BD‐FDRs as compared to controls. Given that a larger ICV is associated with a higher IQ in healthy individuals, these findings suggest that the larger ICV in BD‐FDRs is unrelated to differences in IQ (which was nonsignificantly lower in BD‐FDRs compared to controls). Future work could compare the finding of ICV in risk for bipolar disorder to autism, another neurodevelopmental disorder. Patients with autism have larger ICV compared to controls (Van Rooij et al., 2018), while not having a functional benefit in form of higher intelligence. One could argue the same (to a much lesser extent) is true for the BD‐FDRs. This may add another piece of information to disentangle risk for psychiatric disease, brain deficits and cognition.

Taken together, the study findings provide suggestive evidence for different genetic influences on neurodevelopmental processes in SZ‐FDRs and BD‐FDRs, leading to larger ICV and lower IQ in those at familial risk for bipolar disorder and lower IQ and but similar ICV in those at familial risk for schizophrenia compared to controls.

### 4.4 Limitations

A few limitations to this study should be taken into account. This work is a meta‐analysis of multiple cohorts from research centers around the world. We were therefore not able to perform analyses such as predictive modeling which requires the raw data. One of the main reasons to choose this approach is that the actual imaging data does not have to be shared, which substantially increased our sample size. The study included heterogeneous samples (e.g., in acquisition protocols, scanner field strength, FreeSurfer version, IQ test battery, schooling systems, inclusion and exclusion criteria). Meta‐analysis approaches find consistent effects despite this variance but cannot account for all sources of heterogeneity. One source of heterogeneity might also be the substantial age differences between the different cohorts. Both adult and children/adolescent cohorts were included in the analyses, and considering that the brains of the children and adolescents have not reached its adults size and that they have not yet reached the average age‐at‐onset, might have influenced the findings of the overall effects. In addition, the FDR groups consist of multiple first‐degree relative types (parents, siblings, offspring, co‐twins). We decided not analyze each relative type separately, as our prior study showed insufficient power to detect group differences between the different relatives subtypes (de Zwarte, Brouwer, Agartz, et al., 2019). Importantly, the composition of the SZ‐FDRs and BD‐FDRs groups differed. More SZ‐FDRs were included, of whom a larger proportion were siblings, whereas there were more offspring in the BD‐FDRs group. This indicates an overall systematic difference in the way bipolar and schizophrenia families were recruited and highlights that these are not epidemiologically acquired samples representing the entire population of relatives. This could confound the differences reported in the SZ‐FDRs and BD‐FDRs. Finally, we only analyzed current IQ and educational attainment as cognitive measures in relation to brain structure. Little to no information was available in the participating cohorts on some demographic features, such as parental socioeconomic status (SES), longitudinal cognitive performance (to address cognitive development over time) and other environmental factors that are potentially related to brain structure and to risk for schizophrenia and/or bipolar disorder.

## 5 CONCLUSIONS

In summary, investigating family members of patients with schizophrenia and bipolar disorder can provide insight into the effect of familial risk of these disorders on the brain and cognition. This study showed differential global cortical thickness and surface area abnormalities in SZ‐FDRs and BD‐FDRs. While present in both relative groups, cognitive alterations were more pronounced in SZ‐FDRs, adding to the evidence that cognition is more affected in (risk for) schizophrenia than in (risk for) bipolar disorder. Brain differences in the relatives were related to cognitive alterations, as expected based on the well‐established positive relationship between intelligence and brain. However, we found no evidence that the larger ICV in BD‐FDRs was related to IQ, nor were differences in other brain measures between relatives and controls explained by IQ. This suggests that differential brain developmental trajectories underlying predisposition to schizophrenia or bipolar disorder are only minimally related to IQ. This study of schizophrenia and bipolar disorder relatives further disentangles the biological underpinnings of both disorders. The resulting findings may also inform the ongoing debate on whether schizophrenia and bipolar disorder should be conceptualized as different categories or whether they are part of a continuum of symptoms.

## CONFLICT OF INTEREST

Dr Yalin has been an investigator in clinical studies conducted together with Janssen‐Cilag, Corcept Therapeutics, and COMPASS Pathways in the last 3 years. Dr Cannon reports that he is a consultant to Boerhinger Ingelheim Pharmaceuticals and Lundbeck A/S. Dr Meyer‐Lindenberg has received consultant fees from Boehringer Ingelheim, BrainsWay, Elsevier, Lundbeck International Neuroscience Foundation, and Science Advances. Drs Ching, Jahanshad, and Thompson received partial research support from Biogen, Inc. (Boston, MA) for work unrelated to the topic of this manuscript. Dr Vieta has received grants and served as consultant, advisor or CME speaker for the following entities (work unrelated to the topic of this manuscript): AB‐Biotics, Abbott, Allergan, Angelini, Dainippon Sumitomo Pharma, Galenica, Janssen, Lundbeck, Novartis, Otsuka, Sage, Sanofi‐Aventis, and Takeda. The remaining authors report no biomedical financial interests or potential conflicts of interest.

## Supporting information


**Figure S1** Cohen's *d* effect sizes comparing bipolar patients, bipolar relatives, schizophrenia patients, and schizophrenia relatives to controls on (a) regional cortical thickness, (b) only cortical thickness regions surviving false discovery rate correction for multiple testing (*q* < 0.05)
**Figure S2**. Cohen's *d* effect sizes comparing bipolar patients, bipolar relatives, schizophrenia patients, and schizophrenia relatives to controls on (a) regional cortical thickness corrected for mean thickness, (b) only cortical thickness regions corrected for mean thickness surviving false discovery rate correction for multiple testing (*q* < 0.05)
**Figure S3**. Cohen's *d* effect sizes comparing bipolar patients, bipolar relatives, schizophrenia patients, and schizophrenia relatives to controls on (a) regional cortical surface area, (b) only cortical surface area regions surviving false discovery rate correction for multiple testing (*q* < 0.05)
**Figure S4.** Cohen's *d* effect sizes comparing bipolar patients, bipolar relatives, schizophrenia patients, and schizophrenia relatives to controls on (a) regional cortical surface area corrected for total surface area, (b) only cortical surface area regions corrected for total surface area surviving false discovery rate correction for multiple testing (*q* < 0.05)
**Figure S5**. Cohen's *d* effect sizes comparing bipolar patients (light blue), bipolar relatives (blue), schizophrenia patients (pink), and schizophrenia relatives (red) to controls on (a) global brain measures, corrected for (b) intracranial volume (ICV), (c) intelligent quotient (IQ), (d) educational attainment. The error bars depict the lower and upper 95% confidence intervals (CIs).
**Figure S6**. Cohen's d effect sizes comparing bipolar patients (light blue), bipolar relatives (blue), schizophrenia patients (pink), and schizophrenia relatives (red) to controls on (a) subcortical volumes, corrected for (b) intracranial volume (ICV), (c) intelligent quotient (IQ), (d) educational attainment. The error bars depict the lower and upper 95% confidence intervals (CIs).

**Table S1** Sample inclusion criteria
**Table S2.** IQ test battery description, NA, not applicable
**Table S3.** Educational attainment (i.e., years of education completed) criteria description, NA, not applicable
**Table S4.** Sample image acquisition and image processing details
**Table S5.** Cohen's *d* effect size bipolar and schizophrenia relatives, controlled for IQ (middle column) and controlled for educational attainment (right column)
**Table S6.** Cohen's *d* effect size bipolar and schizophrenia patients, controlled for IQ (middle column) and controlled for educational attainment (right column)
**Table S7.** Cohen's *d* effect size bipolar and schizophrenia relatives corrected for ICV (except ICV, SA, and CT), controlled for IQ (middle column) and controlled for educational attainment (right column)
**Table S8.** Cohen's *d* effect size bipolar and schizophrenia patients corrected for ICV (except ICV, SA, and CT), controlled for IQ (middle column) and controlled for educational attainment (right column)
**Table S9.** Correlations brain and IQ; across all subjects

## Data Availability

The data that support the findings of this study are available from the corresponding author upon reasonable request.
