# Supplementary material for: Intelligence, educational attainment, and brain structure in those at familial high‐risk for schizophrenia or bipolar disorder
Source: Hum Brain Mapp. 2020 Oct 7;43(1):414–30. doi: 10.1002/hbm.25206 (PMC8675411; doi:10.1002/hbm.25206)
Supplement: Supplementary file 1 — Figure S1 Cohen's d effect sizes comparing bipolar patients, bipolar relatives, schizophrenia patients, and schizophrenia relatives to controls on (a) regional cortical thickness, (b) only cortical thickness regions surviving false discovery rate correction for multiple testing (q < 0.05) Figure S2. Cohen's d effect sizes comparing bipolar patients, bipolar relatives, schizophrenia patients, and schizophrenia relatives to controls on (a) regional cortical thickness corrected for mean thickness, (b) only cortical thickness regions corrected for mean thickness surviving false discovery rate correction for multiple testing (q < 0.05) Figure S3. Cohen's d effect sizes comparing bipolar patients, bipolar relatives, schizophrenia patients, and schizophrenia relatives to controls on (a) regional cortical surface area, (b) only cortical surface area regions surviving false discovery rate correction for multiple testing (q < 0.05) Figure S4. Cohen's d effect sizes comparing bipolar patients, bipolar relatives, schizophrenia patients, and schizophrenia relatives to controls on (a) regional cortical surface area corrected for total surface area, (b) only cortical surface area regions corrected for total surface area surviving false discovery rate correction for multiple testing (q < 0.05) Figure S5. Cohen's d effect sizes comparing bipolar patients (light blue), bipolar relatives (blue), schizophrenia patients (pink), and schizophrenia relatives (red) to controls on (a) global brain measures, corrected for (b) intracranial volume (ICV), (c) intelligent quotient (IQ), (d) educational attainment. The error bars depict the lower and upper 95% confidence intervals (CIs). Figure S6. Cohen's d effect sizes comparing bipolar patients (light blue), bipolar relatives (blue), schizophrenia patients (pink), and schizophrenia relatives (red) to controls on (a) subcortical volumes, corrected for (b) intracranial volume (ICV), (c) intelligent quotient (IQ), (d) educational attainment. The e [file HBM-43-414-s001.docx]

SUPPLEMENTARY FIGURES

a)


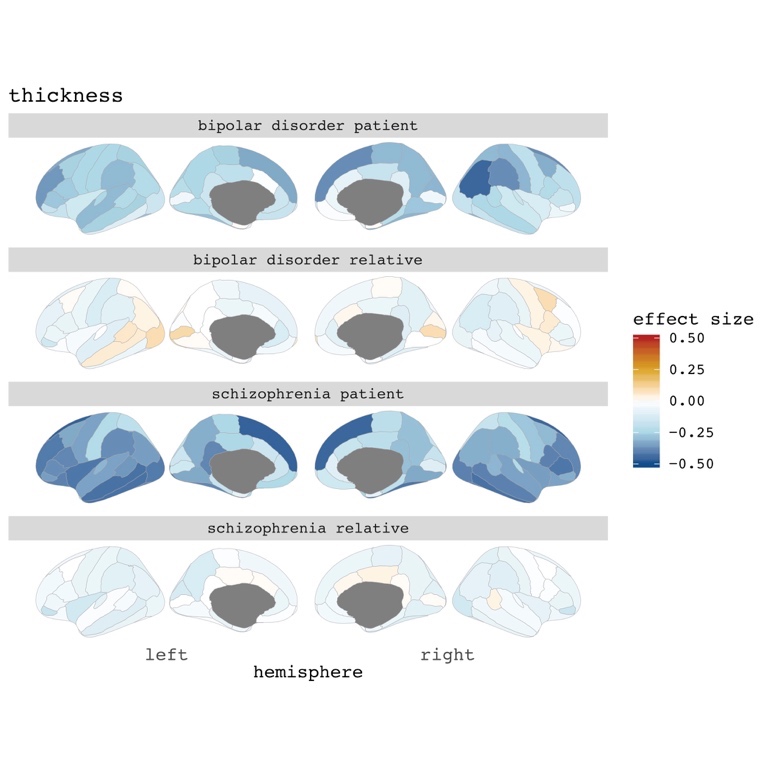


b)


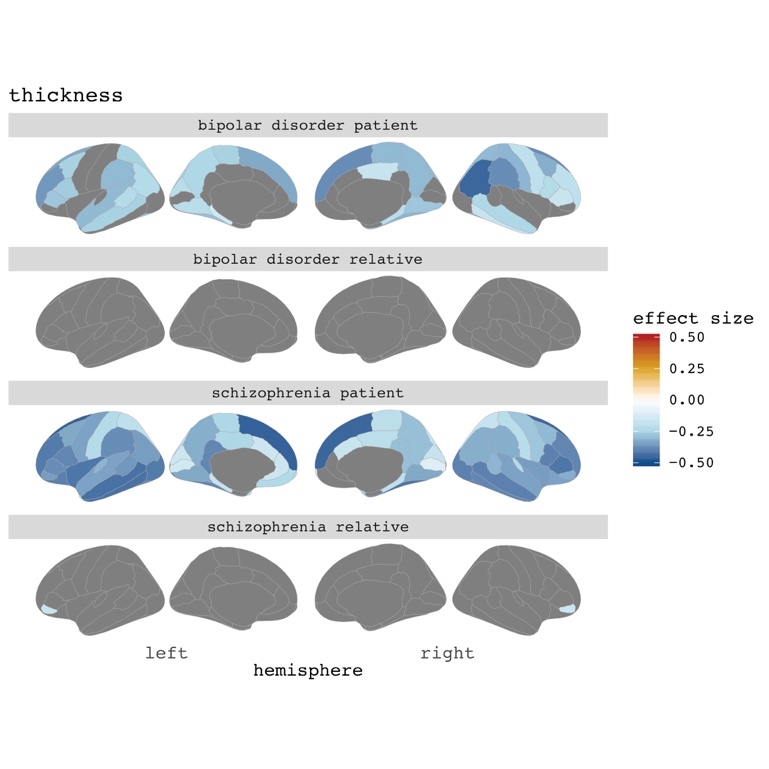


**Supplementary Figure 1**. Cohen’s *d* effect sizes comparing bipolar patients, bipolar relatives, schizophrenia patients, and schizophrenia relatives to controls on a) regional cortical thickness, b) only cortical thickness regions surviving false discovery rate correction for multiple testing (*q* < 0.05)

a)


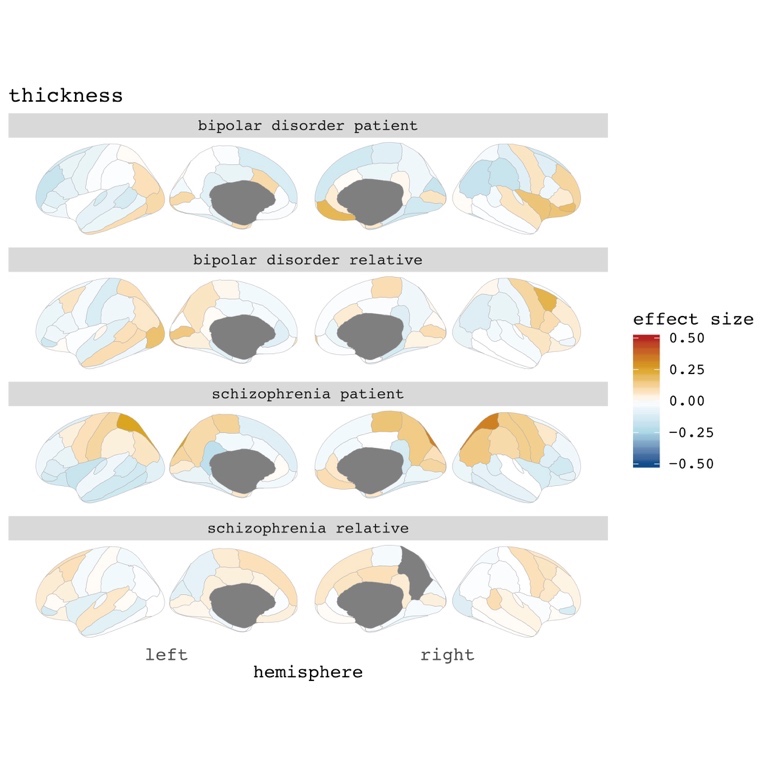


b)


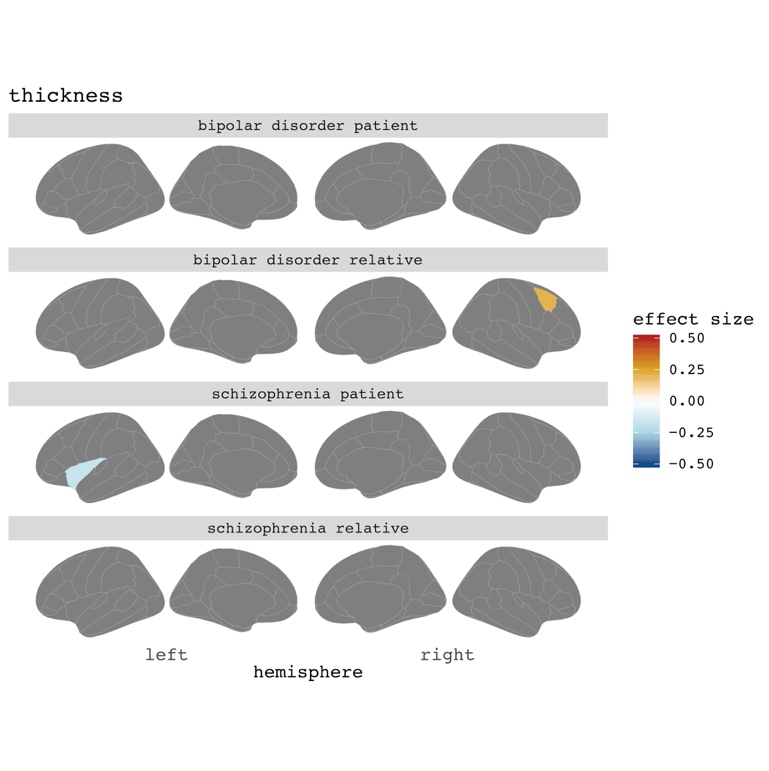


**Supplementary Figure 2**. Cohen’s *d* effect sizes comparing bipolar patients, bipolar relatives, schizophrenia patients, and schizophrenia relatives to controls on a) regional cortical thickness corrected for mean thickness, b) only cortical thickness regions corrected for mean thickness surviving false discovery rate correction for multiple testing (*q* < 0.05)

a)


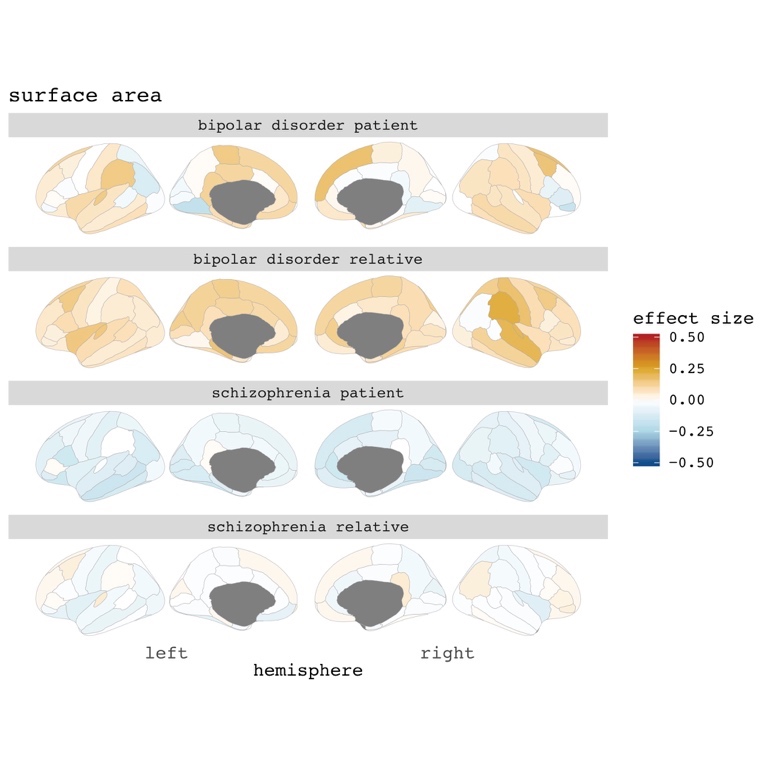


b)


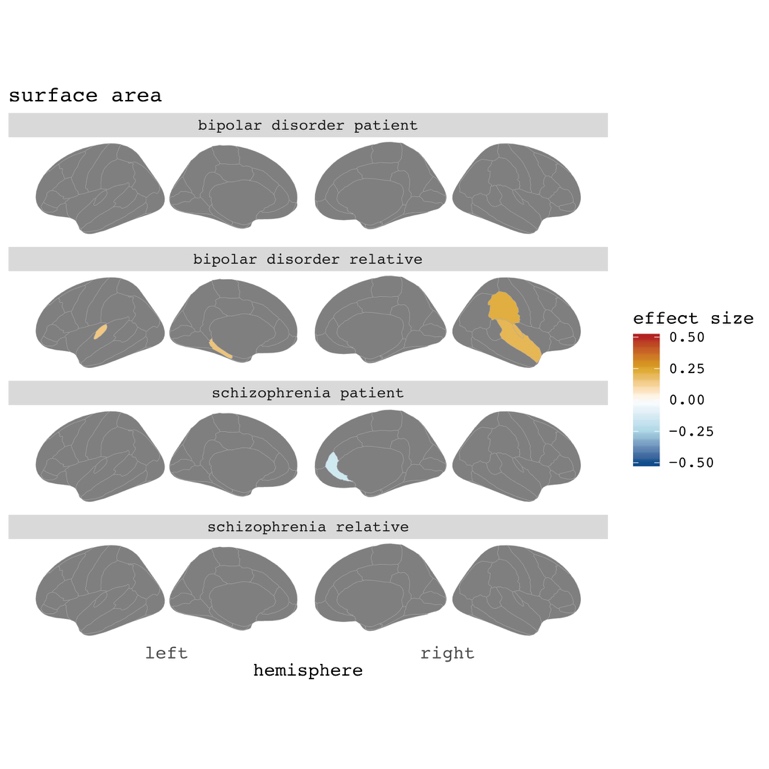


**Supplementary Figure 3**. Cohen’s *d* effect sizes comparing bipolar patients, bipolar relatives, schizophrenia patients, and schizophrenia relatives to controls on a) regional cortical surface area, b) only cortical surface area regions surviving false discovery rate correction for multiple testing (*q* < 0.05)

a)


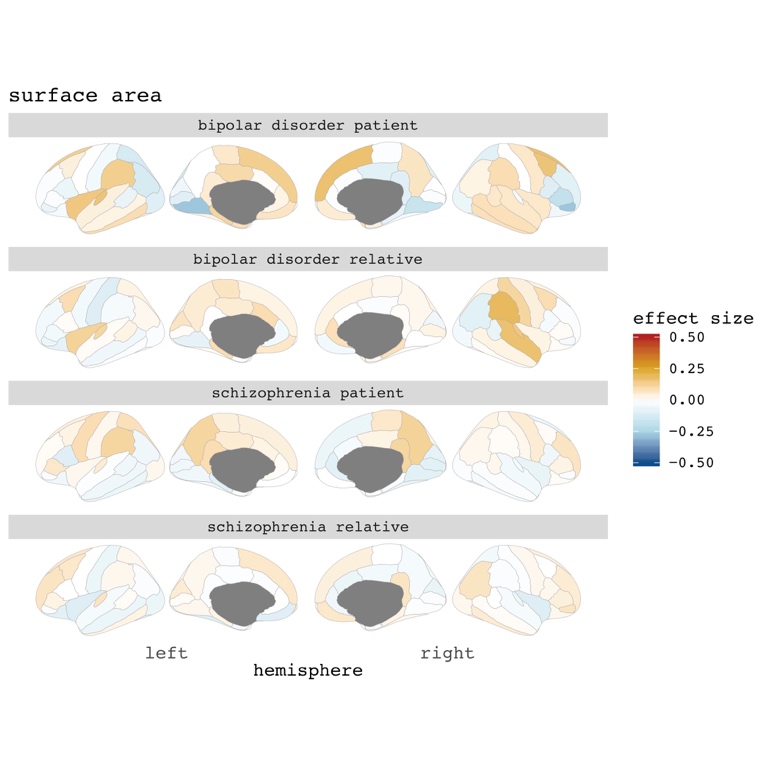


b)


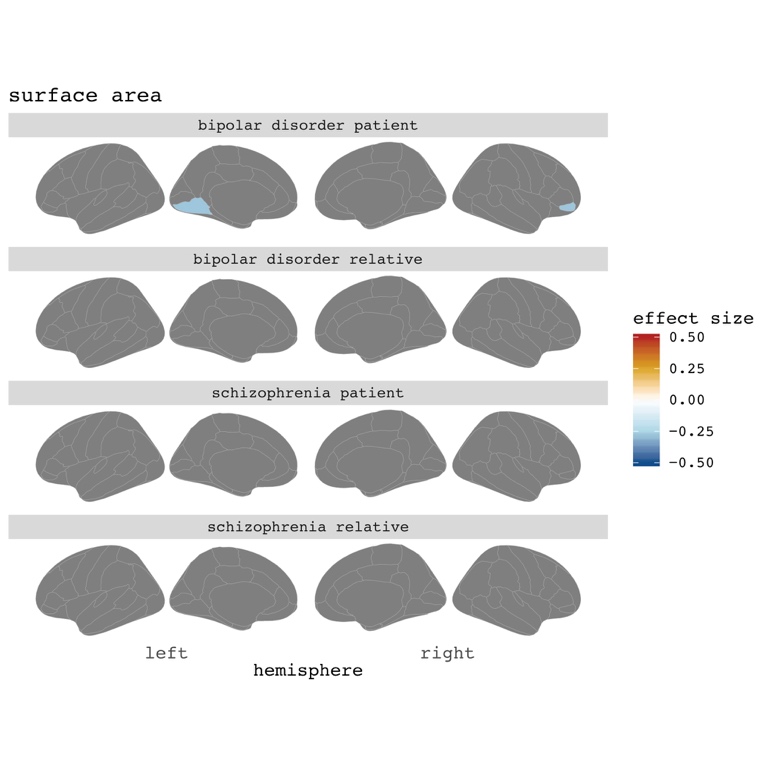


**Supplementary Figure 4.** Cohen’s *d* effect sizes comparing bipolar patients, bipolar relatives, schizophrenia patients, and schizophrenia relatives to controls on a) regional cortical surface area corrected for total surface area, b) only cortical surface area regions corrected for total surface area surviving false discovery rate correction for multiple testing (*q* < 0.05)

a) b)


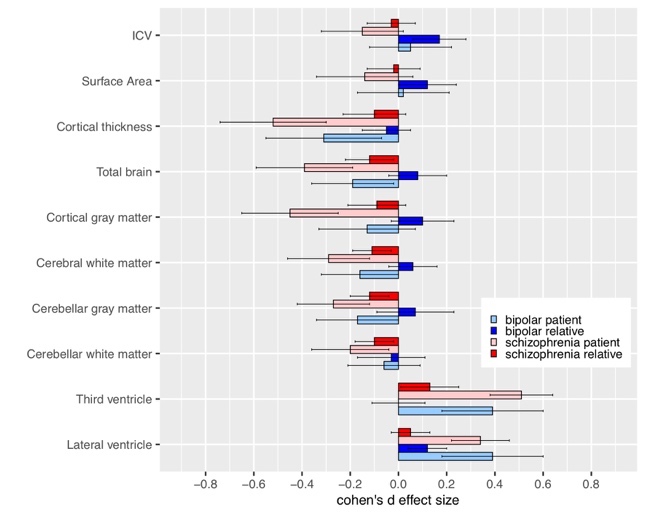

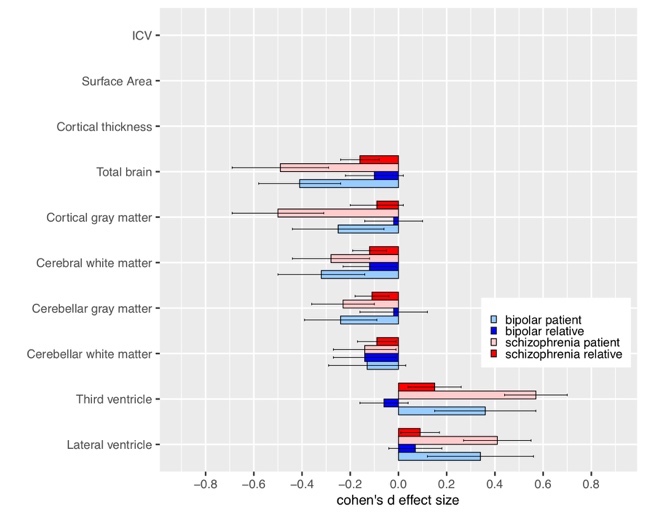


c) d)


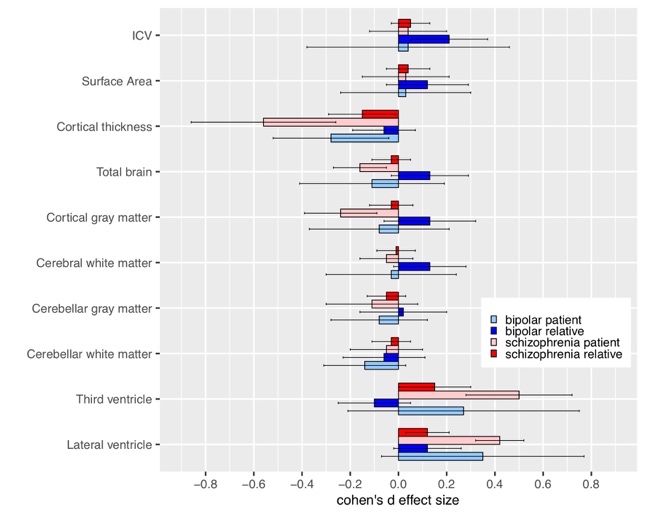

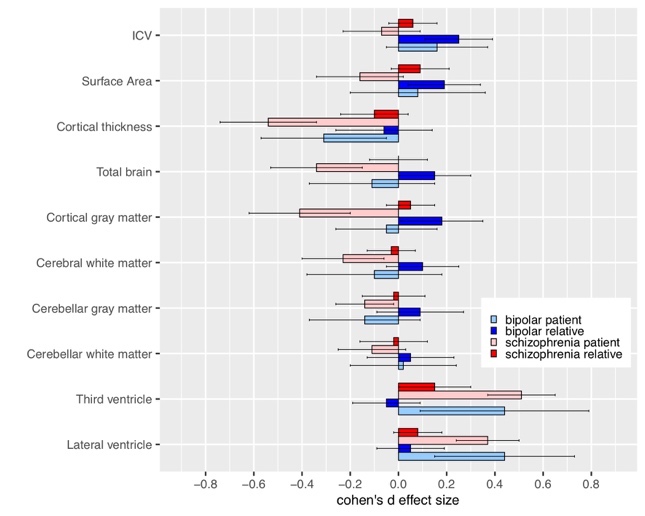


**Supplementary Figure 5**. Cohen’s *d* effect sizes comparing bipolar patients (light blue), bipolar relatives (blue), schizophrenia patients (pink), and schizophrenia relatives (red) to controls on a) global brain measures, corrected for b) intracranial volume (ICV), c) intelligent quotient (IQ), d) educational attainment. The error bars depict the lower and upper 95% confidence intervals (CIs).

a) b)


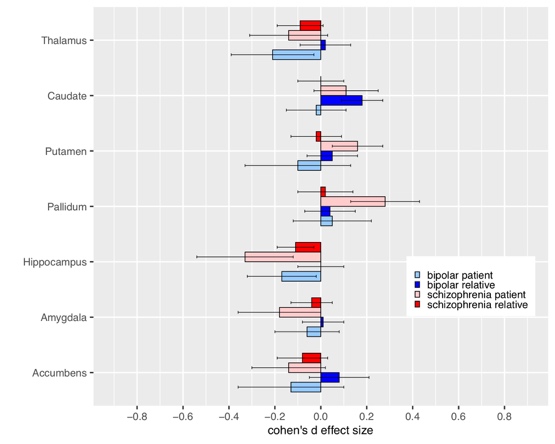

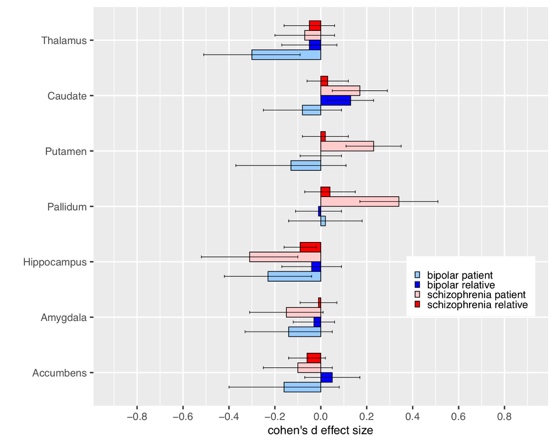


c) d)


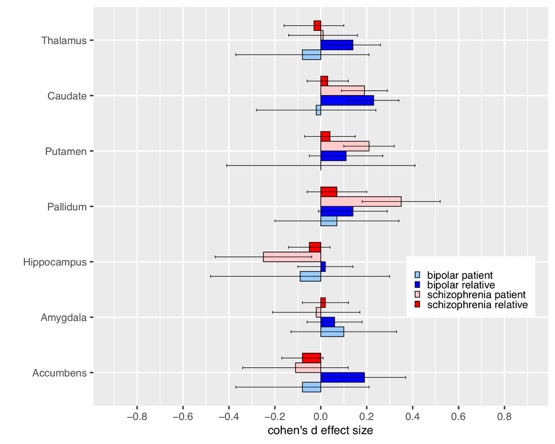

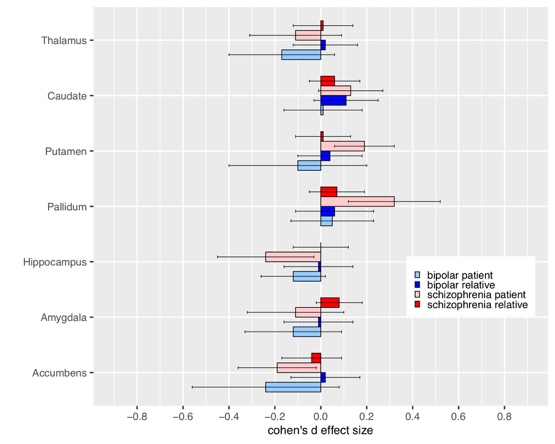


**Supplementary Figure 6**. Cohen’s d effect sizes comparing bipolar patients (light blue), bipolar relatives (blue), schizophrenia patients (pink), and schizophrenia relatives (red) to controls on a) subcortical volumes, corrected for b) intracranial volume (ICV), c) intelligent quotient (IQ), d) educational attainment. The error bars depict the lower and upper 95% confidence intervals (CIs).
