# Supplementary material for: Intelligence, educational attainment, and brain structure in those at familial high‐risk for schizophrenia or bipolar disorder
Source: Hum Brain Mapp. 2020 Oct 7;43(1):414–30. doi: 10.1002/hbm.25206 (PMC8675411; doi:10.1002/hbm.25206)
Supplement: Supplementary file 2 — Table S1 Sample inclusion criteria Table S2. IQ test battery description, NA, not applicable Table S3. Educational attainment (i.e., years of education completed) criteria description, NA, not applicable Table S4. Sample image acquisition and image processing details Table S5. Cohen's d effect size bipolar and schizophrenia relatives, controlled for IQ (middle column) and controlled for educational attainment (right column) Table S6. Cohen's d effect size bipolar and schizophrenia patients, controlled for IQ (middle column) and controlled for educational attainment (right column) Table S7. Cohen's d effect size bipolar and schizophrenia relatives corrected for ICV (except ICV, SA, and CT), controlled for IQ (middle column) and controlled for educational attainment (right column) Table S8. Cohen's d effect size bipolar and schizophrenia patients corrected for ICV (except ICV, SA, and CT), controlled for IQ (middle column) and controlled for educational attainment (right column) Table S9. Correlations brain and IQ; across all subjects [file HBM-43-414-s002.docx]

SUPPLEMENTARY TABLES

**Supplementary Table 1.** Sample inclusion criteria

| Sample | Inclusion criteria |
| --- | --- |
| BPO-FLB | BD patients were diagnosed with either type I or type II BD (BD I and BD II), or BD not otherwise specified (BD NOS) according to DSM-IV. Patients exclusion criteria included substance use within the past six months and general medical problems. Inclusion criteria for offspring of BD patients included diagnosis of BD in biological father and or mother according to SCID. Inclusion criteria for healthy controls included those without a history of any psychiatric/neurological disorders or mood disorders in first degree relatives. Exclusion criteria for patient, healthy control and offspring groups included head injury with loss of consciousness, presence of metallic objects in the body, family history of hereditary neurological disorders, and pregnancy. |
| C-SFS | Schizophrenia and schizoaffective patients participated. Inclusion criteria for all participants included: (1) age 18-65; (2) minimum intelligence quotient (IQ) of 70 as measured by Wechsler Abbreviated Scale of Intelligence; (3) no current diagnosis of drug or alcohol dependence or abuse; (4) no history of head injury or being unconscious for more than 20 minutes; (5) no history of electroconvulsive therapy; and (6) no history of a neurological condition. Further criteria for inclusion of relatives and controls were no lifetime diagnosis of a psychotic or bipolar disorder, Axis II Cluster A disorder, or history of anti-psychotic medication use. Further criterion for inclusion of community controls was no family history of a psychotic or bipolar disorder. |
| Cardiff | All participants were age 35 years or older and included: (1) individuals with confirmed diagnosis of bipolar disorder type I or type II, euthymic at time of recruitment and reporting mood stability and no-change in medication for 1 month prior scanning; (2) unaffected relatives of bipolar participants with no personal history of mood disorders or psychosis; (3) healthy controls with no personal or first-degree family history of mental disorders. All DSM-IV diagnoses were confirmed through the Mini-international neuropsychiatric interview (Sheehan et al, 1998). Patients were recruited through the Bipolar Disorder Research Network (BDRN) and the National Centre for Mental Health (NCMH) both at Cardiff University, non-affected siblings were recruited via BD participants, and healthy controls from the community via advertisement. |
| CLiNG – BD | Inclusion criteria for participants were a) age between 18 and 60 years, b) parents, siblings or offspring of index patients with bipolar disorder, c) no own diagnosis of a mental disorder and d) right-handedness. Diagnosis of bipolar disorder in index patients was made by an experienced clinician using the German version of the Structured Clinical Interview for DSM-IV, unless a medical report confirming diagnosis of bipolar disorder was provided. Exclusion criteria included history of neurological and severe medical disorders, current or past psychopathology as well as substance dependence and substance abuse. |
| CLiNG – SZ | Inclusion criteria for participants were a) age between 18 and 60 years, b) parents, siblings or offspring of index patients with schizophrenia, c) no own diagnosis of a mental disorder and d) right-handedness. Diagnosis of schizophrenia in index patients was made by an experienced clinician using the German version of the Structured Clinical Interview for DSM-IV, unless a medical report confirming diagnosis of schizophrenia was provided. Exclusion criteria included history of neurological and severe medical disorders, current or past psychopathology as well as substance dependence and substance abuse. |
| DEU | The inclusion criteria for patient group were having a diagnosis of bipolar disorder type I according to DSM-IV, aging between 18 and 65 years, being in euthymic state (according to DSM-IV and scoring ≤7 on both Young Mania Rating Scale and Hamilton Rating Scale for Depression) for at least six months and having no axis I comorbidity. The inclusion criteria for first degree relatives of bipolar disorder patients were having no lifetime axis I diagnosis, and for healthy controls, having no lifetime axis I diagnosis and family history for psychiatric disorders at the time of recruitment. The following exclusion criteria were applied to all groups: presence of auditory or visual impairment, history of neurosurgical intervention, being pregnant or breastfeeding, diagnosis of neurocognitive illness or substance use during the preceding six weeks before participating in the study. All participants were evaluated using the Structured Clinical Interview for Diagnostic Statistical Manual-IV (DSM-IV) (SCID-I). |
| EGEU | All participants were aged between 20 and 55 years old and included: (1) patients with bipolar disorder type 1, euthymic at the time of recruitment (defined as scoring less than five on the Young Mania Rating Scale (YMRS), and less than 11 on the Hamilton Depression Rating Scale-17 item (HAM-D-17) for at least three months prior to and during the MRI scanning); (2) healthy siblings of bipolar participants, never diagnosed with mental illness; (3) unrelated healthy controls, no personal or family history of mental illness. All patients were recruited from the Ege University School of Medicine’s Department of Psychiatry, where the patients had been receiving follow-up care with monthly assessments for at least three years, healthy siblings were recruited via BD patients, and unrelated healthy controls from community via local advertisement. |
| EHRS | All participants were aged between 16 and 25 years old and recruited across Scotland. High-risk individuals were included if they had no history of serious psychiatric problems and had at least two first- or second-degree relatives affected with schizophrenia. Participants for the control group were recruited from the social network of the high-risk individuals themselves; they had no personal or family history of other psychotic illness, but could have a family history of other psychiatric illness and otherwise were similar to the high-risk participants as possible. First-episode individuals were recruited from local hospitals, were balanced group-wise for age with the high-risk individuals and had no family history of schizophrenia. |
| ENBD_UT | Specific inclusion criteria for the BD sibling pairs are: a) BD proband with diagnosis of BD I or II, based on DSM-IV criteria, b) having a same-gender sibling not affected by BD; c) ages 18-65 years old; d) BD proband and unaffected sibling no more than 10 years apart in age; e) BD proband at any current mood state at the time of the study; f) BD proband preferably off pharmacological treatment at the time of study, but if not feasible, being on antidepressants and mood stabilizers (including anticonvulsants, typical and atypical antipsychotics, and lithium will be allowed; g) BD proband and unaffected sibling brought up together in the same family. Exclusion criteria for the BD sibling pairs: a) diagnosis of Bipolar Disorder, Schizoaffective Disorder or Schizophrenia is not allowed. Alcohol and substance abuse/ dependence (if in remission in the past 6 months) and anxiety disorders are allowed; b) being on a regular dose of benzodiazepines within two weeks of study participation; c) pregnancy d) ineligibility or inability of one of the members of the sibling pair to participate in the study. Exclusion criteria for controls: a) a lifetime psychiatric diagnosis, b) family history of psychiatric illness in a first-degree relative. |
| FIDMAG-Clinic | The patients met DSM-IV criteria for bipolar disorder, based on interview and review of case notes, and were euthymic at the time of scanning. The unaffected siblings and healthy controls were excluded if they reported a history of mental illness and/or treatment with psychotropic medication as assessed using the Computerized Diagnostic Interview Schedule for the DSM-IV. Healthy controls were also excluded if they had a first-degree relative with a major psychiatric disorder. Patients, siblings and controls were also excluded if: (a) they were younger than 18 or older than 65 years; (b) they had a history of brain trauma or neurological disease; (c) they had shown alcohol/substance abuse within 12 months prior to participation; or (d) they had undergone electroconvulsive therapy in the previous 12 months. |
| Geneva | The offspring were aged between 15 and 25 years old at inclusion. Proband parent were outpatients from the Geneva University Hospital, followed in the Mood Disorder Unit. BD diagnostic in the proband was established with the Mini‐International Neuropsychiatric Interview (MINI; Hergueta, Baker, & Dunbar, 1998) as part of the standard evaluation. Offspring of BD patients were recruited after their parents gave formal consent to contact their children.Participants younger than 18 years of age were assessed with the French version of the Schedule for Affective Disorders and Schizophrenia for School‐Age Children (K‐SADS; Kaufman et al., 1997). Participants older than 18 years of age were assessed with the French version of the Diagnostic Interview for Genetic Studies (DIGS; Preisig et al., 1999).Control subjects were matched for age, gender, laterality, and years of education, and were recruited through advertisements placed at the University of Geneva and on classified web sites. Inclusion criteria for controls were age, no history of psychiatric or neurological treatment for the subjects, and no reported history of a psychiatric disorder for their parents, as assessed during the interview of the subject. All participants gave written informed consent before assessment. The research was conducted according to the principles of the Declaration of Helsinki and was approved by the University of Geneva research ethics committee (CER 13–081). |
| HUBIN | Patients diagnosed with long term psychotic disorder were recruited from outpatient clinics in the North-Western part of Stockholm County. The patients were diagnosed according to DSM-III-R and DSM-IV based on information from interviews and medical records. Non-psychotic siblings of patients with psychosis were asked to participate when their relative with a psychotic disorder had agreed to their participation. Control subjects were recruited among students, hospital staff members or from a population register. All controls with the exception of those recruited from a population register had earlier attended in biological research at the Karolinska Institute (Jönsson et al., 2006, Laywer et al., 2006, Nesvåg et al., 2008). The controls consisted of non-psychotic individuals unrelated to the patients. Neither the siblings, nor the controls received any psychotic diagnosis according to DSM-III-R and DSM-IV. |
| IDIBAPS | The study was conducted in the Child and Adolescent Psychiatry Department of the Hospital Clinic of Barcelona, Spain. The protocol was approved by the local ethics review board and further details of the sample can be found in Sanchez-Gistau et al., 2015 (Schizophrenia Research 168 (2015) 197–203). Patients with a diagnosis of schizophrenia or bipolar disorder from adult psychiatry units with offspring 6 to 17 years old were identified and invited to participate in the study. The exclusion criteria for proband parents were intellectual disability and drug or medically induced psychosis or mania. Exclusion criteria for offspring included intellectual disability, head injury with loss of consciousness, or severe neurological conditions. Community control parents were recruited through advertisements posted in primary health care centers and other community locations within the same geographic area as the patients. The exclusion criteria were intellectual disability, severe neurological conditions and personal or first-degree family history of schizophrenia or bipolar spectrum disorders. All 6- to 17-year-old offspring of community control parents were invited to participate in the study; exclusion criteria were the same as those for high-risk offspring. To decrease selection bias, parents who stated they were specifically motivated to participate because of concerns about school performance or emotional or behavioral problems in their offspring were excluded. |
| IoP – BD | Twins were recruited using a variety of methods, these were: 1. Direct contact with health professionals, including psychiatrists, clinical psychologists, occupational therapists and so on; 2. Advertising: Adverts were placed national and local newspapers as well as in specific user group publications such as Pendulum, the Manic Depression Fellowship’s quarterly newsletter. Flyers for the study were also distributed in hospitals, clinics and chemists. Links were also placed on various internet sites such as Wikipedia.org and self-help groups; and 3. Talks were given by team members at service user and professional conferences. Control subjects were recruited primarily via advertising in the national media, with further recruitment from a pool of research participants obtained for previous studies conducted at the Institute of Psychiatry (IoP, now IoPPN), with a smaller group being referred by members of staff at the Bethlem and Maudsley Hospital Trust and word of mouth. Exclusion criteria for all participants were a history of neurologic illness or of systemic illness with known neurologic complication, history of head injury with loss of consciousness, and current substance misuse or dependence. Controls had no personal or family history of psychotic illness. Controls and unaffected relatives with a nonpsychotic psychiatric diagnosis were included. All participants were between 16 and 65 years-old at the time of participation. All the studies were approved by institutional review boards, and all the participants gave written informed consent before participating. Further information can be found in Georgiades et al. (2016) and Sugihara et al. (2017). |
| IoP – SZ | Twins were referred from across the United Kingdom by their treating psychiatrists. Control twins were recruited from the Institute of Psychiatry Volunteer Twin Register and by national media advertisements. Families were referred from clinics and voluntary organizations across the United Kingdom. Control subjects were ascertained from a pool of research participants obtained for previous studies conducted at the Institute of Psychiatry, from members of staff at the Bethlem and Maudsley Hospital Trust, and through advertisements in the press. Exclusion criteria for all participants were a history of neurologic illness or of systemic illness with known neurologic complication, history of head injury with loss of consciousness, and current substance misuse or dependence. Controls had no personal or family history of psychotic illness. Controls and unaffected relatives with a nonpsychotic psychiatric diagnosis were included. All the studies were approved by institutional review boards, and all the participants gave written informed consent before participating. Further information can be found in the following papers (Toulopoulou et al. 2004; Toulopoulou et al. 2003; Toulopoulou et al. 2007; Ettinger et al. 2007; Picchioni et al. 2010). Demographic information including years of education was collected using a standardised interview. With regard to educational achievement, both total years of completed education and highest completed academic qualification were recorded. |
| LIBD | Participants were recruited nationwide as part of a study at the National Institute of Mental Health, Bethesda, MD. Samples used in this study were under a standard procedure including a structured diagnostic interview (Structured Clinical Interview for DSM-IV) and a formal neurological examination. All patients met DSM-IV criteria for schizophrenia or related diagnoses including schizoaffective disorder, psychosis (not otherwise specified), and schizoid, paranoid, and schizotypal personality disorders. The majority of patients were taking antipsychotic medication at the time of scan, and a minority had a lifetime history of comorbid mental illness or substance abuse/dependence (including alcohol). Exclusion criteria for normal controls included a current or past history of neurological or psychiatric disorders, hypertension or drug abuse. A minority of siblings had a past lifetime history of a non-psychotic mental illness and/or substance abuse and/or dependence (39.7%), but none met criteria at the time of evaluation. No subjects in any group had a current history of alcohol or substance abuse within 6 months of being scanned. All subjects provided written informed consent, and participated according to the guidelines of the National Institute of Mental Health Institutional Review Board. |
| Maastricht – GROUP | Participants were recruited in selected representative geographical areas in the Netherlands and Belgium, patients were identified through representative clinicians providing health care for patients with psychotic disorder. Siblings were contacted through participating patients. Mailings and advertisements were effectuated in local newspapers of the same geographical area in order to recruit control participants. Inclusion criteria were; age range 16-50 years, fluent in Dutch language and for patients: a diagnosis of non-affective psychotic disorder with illness duration of <10 years. Siblings and controls were excluded if they had a lifetime diagnosis of any non-affective psychotic disorder. In addition, controls were excluded if they had a first-degree relative with a lifetime diagnosis of any psychotic disorder. This was assessed using the Family Interview for Genetic Studies (FIGS) (Maxwell, 1992). Diagnosis was based on the Diagnostic and Statistical Manual of Mental Disorder-IV (DSM-IV) criteria (APA, 2000), measured with the Comprehensive Assessment of Symptoms and History (CASH) interview (Andreasen et al., 1992). All participants were screened before MRI scanning and excluded based on the following: brain injury with unconsciousness of > than 1 hour, meningitis or other neurological diseases with possible impact on brain structure or function, cardiac arrhythmia requiring medical treatment and severe claustrophobia. Participants with metal corpora aliena were excluded from the study, as were women with intrauterine device status and (suspected) pregnancy. |
| MFS | All individuals were aged 16-70. Participant groups included (i) patients with DSM-IV confirmed diagnoses of schizophrenia or bipolar 1 disorder; (ii) unaffected first-degree relatives of these patients including parents, siblings and offspring; (iii) healthy volunteers with no personal or family history of psychotic illness (McDonald et al, Neuropsych Genetics 2002; McDonald et al, Am J Psychiatry 2006). Families were recruited through voluntary organizations or by direct psychiatric referral and on the basis of either being multiply affected, where the index patient had one or more first- or second- degree relatives with a psychotic disorder, or singly-affected where there was no known family history of psychotic disorder. All of the bipolar disorder patients and relatives were from multiply affected families. Exclusion criteria for all participants included organic brain disease, head trauma resulting in loss of consciousness for more than 5 minutes, or DSM-IV substance or alcohol dependence in the 12 months before the assessment. |
| MooDS – BD | Participants were aged between 18 and 53 years. First degree relatives were offspring or siblings of index patients with BPD. Diagnosis of BPD in index patients was made by an experienced clinician using the German version of the Structured Clinical Interview for DSM-IV, or the patients provided a medical report confirming diagnosis of BPD. All participants had no history of any neurologic disorder or current psychiatric Axis I disorder including drug or alcohol dependence as verified by the nonpatient version of the Structured Clinical Interview for DSM-IV and had no MRI contraindications. |
| MooDS – SZ | Participants were aged between 18 and 55 years. First degree relatives were parents, offspring or siblings of index patients with SCZ. Diagnosis of SCZ in index patients was made by an experienced clinician using the German version of the Structured Clinical Interview for DSM-IV, or the patients provided a medical report confirming diagnosis of SCZ. All participants had no history of any neurologic disorder or current psychiatric Axis I disorder including drug or alcohol dependence as verified by the nonpatient version of the Structured Clinical Interview for DSM-IV and had no MRI contraindications. |
| MSSM | All participants were aged 18 to 67 years. The eligibility criteria for all participants were (a) IQ>70; (b) no history of head trauma or loss of consciousness; (c) no current or lifetime history of medical or neurological disorders; (d) no lifetime history of substance use disorder; (e) no MRI contraindications (e.g. metal implants, claustrophobia). Patients were required to fulfil diagnostic DSM-IV criteria for BD type-1 or type II, while healthy volunteers were included if they had no lifetime personal history of mental disorders and no family history (up to second-degree relatives) of BD. Unaffected relatives of bipolar participants were included if they had no personal history of bipolar disorder or psychosis. |
| Olin | Patients with bipolar I disorder, their unaffected siblings, and unrelated healthy volunteers were recruited from psychiatric facilities and community advertisements in Hartford, Conn. Patients were included if they met DSM-IV criteria for bipolar I disorder based on the Structured Clinical Interview for DSM-IV disorders; had no history of major medical or neurological conditions (e.g. epilepsy, migraine, head trauma with loss of consciousness); had an IQ > 80 (based on WASI); and had a sibling willing to participate in the study. Eligibility criteria for siblings and unrelated healthy volunteers were identical to those for patients, with the exception of a personal lifetime diagnosis of bipolar or psychosis spectrum disorders (having a DSM-IV diagnosis other than bipolar or psychosis spectrum disorders was not an exclusion criterium). In addition, unrelated healthy volunteers could not have a family history of mood or psychotic disorders. All participants provided informed consent as approved by the institutional review board at Hartford Hospital and Yale University. |
| ORBIS I | Participants were recruited from an ongoing Offspring Risk for BD Imaging Study–ORBIS. We recruited offspring from families of well-characterized adult BD probands who had participated in previous genetic and HR studies (Duffy et al., 2002; Lopez de Lara et al., 2010; Hajek et al., 2015b) in Halifax, Nova Scotia. The inclusion criterion was 15–30 years of age. We included participants with BD type I or type II, but not with BD NOS as probands for this study. The offspring from BD probands were divided into two subgroups. (1) The unaffected HR group, which included offspring without a personal history of Axis I psychiatric disorders. These individuals were considered HR because they came from multiplex families (more than one member affected with BD) and had one parent affected with a primary mood disorder. (2) The affected familial group, which included offspring meeting criteria for a lifetime Axis I diagnosis of mood disorders (i.e. a personal history of at least one episode of depression, hypomania, or mania meeting full DSM-IV criteria) and had one parent affected with a primary mood disorder. Depressive episodes were included because unipolar depression is characteristically the first manifestation of illness in patients who later develop BD. Lastly, we recruited control participants free of personal or family history of DSM-IV Axis I psychiatric disorders. Common exclusion criteria for all groups were a personal history of (1) any serious medical or neurologic disorders, (2) substance abuse/dependence during the previous 6 months, or (3) magnetic resonance imaging (MRI) exclusion criteria. |
| ORBIS II | Families were identified through adult probands with BD, who had participated in the Czech Bipolar Disorder Case Registry. Only the offspring from these families, not the probands, were a part of the MRI study. The inclusion criterion was 15–30 years of age. We included participants with BD type I or type II, but not with BD NOS as probands for this study. The offspring from BD parents were divided into two subgroups: 1) the Unaffected HR group, which consisted of offspring with no lifetime history of psychiatric disorders. These individuals were at an increased risk for BD because they had one parent affected with a primary mood disorder. 2) The Affected Familial group, which consisted of offspring who met criteria for a lifetime Axis I diagnosis of mood disorders (i.e., a personal history of at least one episode of depression, hypomania, or mania meeting full DSM-IV criteria). Also, we recruited control participants free of personal or family history of DSM-IV Axis I psychiatric disorders. Common exclusion criteria for all groups were a personal history of (1) any serious medical or neurologic disorders, (2) substance abuse/dependence during the previous 6 months, or (3) magnetic resonance imaging (MRI) exclusion criteria. |
| PENS | Participants with schizophrenia, schizoaffective disorder, or bipolar disorder I, as well as first-degree relatives of individuals with schizophrenia, schizoaffective disorder, or bipolar disorder, and a group of healthy controls were recruited through the Minneapolis VA, Craigslist, and the community. All participants were aged 18 to 59 years old and underwent a SCID interview to screen for DSM-IV-TR diagnostic criteria, with final diagnostic decisions made through consensus of two trained staff members. For relatives, the diagnosis of the family member with a psychosis spectrum or bipolar disorder was established by our research staff. Psychosis subjects were required to be stable outpatients. Participants without a primary psychotic or bipolar disorder (i.e., relatives and controls) additionally completed the Structured Interview for Schizotypy to rule out Cluster A personality disorders. Exclusion criteria for all subjects included English as a second language, mental retardation (WAIS IQ<70), current alcohol or drug dependence, current or past central nervous system condition, history of electroconvulsive therapy, history of stroke, history of head injury with skull fracture or substantial loss of consciousness (>30 minutes), and all standard MRI contraindications. |
| PHCP | Participants with schizophrenia, schizoaffective disorder, or bipolar disorder I with psychotic features, as well as first-degree relatives of individuals with schizophrenia, schizoaffective disorder, or bipolar disorder, and a group of healthy controls were recruited through the Minneapolis VA, University of Minnesota, and the community. All participants were aged 18 to 69 years old and underwent a SCID interview to screen for DSM-IV-TR diagnostic criteria, with final diagnostic decisions made through consensus of two trained staff members. For relatives, the diagnosis of the family member with a psychosis spectrum disorder was established by our research staff. Psychosis subjects were required to be stable outpatients. Participants without a primary psychotic disorder (i.e., relatives and controls) additionally completed the Structured Interview for Schizotypy to rule out Cluster A personality disorders. |
| STAR (Swedish) BD twin cohort | Subjects were identified on a nation-wide basis through the Sweden Twin Registry. Twin pairs were eligible for inclusion if they were same sex, between the ages of 25 and 65, and born in Sweden between 1940 and 1985 (inclusive). To ascertain twin pairs comprising at least one twin with a diagnosis of schizophrenia or bipolar disorder, this set of twins was screened using hospital admission and discharge diagnosis information from the Swedish National Patient Registry. Monozygotic and dizygotic pairs were recruited from all counties in Sweden and invited to Karolinska Institute for structured diagnostic interviews and additional evaluations, including neuroimaging. Final diagnoses were determined by a consensus procedure. Zygosity was determined for nearly all twin pairs using DNA testing or a well-validated screening measure for those without DNA available on both co-twins. Exclusion criteria were presence of a neurological disorder, history of significant head injury with loss of consciousness, mental retardation, history of substance dependence within 6 months of the screening interview, or inability to read or comprehend spoken and written Swedish. Healthy control pairs were recruited to match proband pairs on age, sex, and zygosity. Healthy controls were excluded if they had a family history of schizophrenia or bipolar disorder according to medical records or self-report. |
| STAR (Swedish) SZ twin cohort | Subjects were identified on a nation-wide basis through the Sweden Twin Registry. Twin pairs were eligible for inclusion if they were same sex, between the ages of 25 and 65, and born in Sweden between 1940 and 1985 (inclusive). To ascertain twin pairs comprising at least one twin with a diagnosis of schizophrenia or bipolar disorder, this set of twins was screened using hospital admission and discharge diagnosis information from the Swedish National Patient Registry. Monozygotic and dizygotic pairs were recruited from all counties in Sweden and invited to Karolinska Institute for structured diagnostic interviews and additional evaluations, including neuroimaging. Final diagnoses were determined by a consensus procedure. Zygosity was determined for nearly all twin pairs using DNA testing or a well-validated screening measure for those without DNA available on both co-twins. Exclusion criteria were presence of a neurological disorder, history of significant head injury with loss of consciousness, mental retardation, history of substance dependence within 6 months of the screening interview, or inability to read or comprehend spoken and written Swedish. Healthy control pairs were recruited to match proband pairs on age, sex, and zygosity. Healthy controls were excluded if they had a family history of schizophrenia or bipolar disorder according to medical records or self-report. |
| SydneyBipolarGroup | All participants were aged between 12 and 30 years and included: (1) individuals with a confirmed diagnosis of bipolar disorder I, II, or schizoaffective disorder; (2) the offspring or siblings of a proband with a confirm DSM-IV diagnosis of bipolar disorder I, II, or schizoaffective disorder; (3) control subjects with no family history of bipolar disorder I or II, schizoaffective disorder, schizophrenia, recurrent major depression, recurrent substance abuse, or psychiatric hospitalisation, and no personal history of bipolar disorder I, II, or schizoaffective disorder. Current or lifetime diagnoses of psychiatric disorders other than bipolar disorder were not considered an exclusion factor for controls or bipolar relatives. All DSM-IV diagnoses were confirmed by two independent raters using Best Estimate Methodology (Leckman JF, Sholomskas D, Thompson WD, Belanger A, & Weissman MM, 1982) and the K-SADS-BP (Nurnberger JI Jr. et al., 2011) or DIGS Version 4 (Nurnberger JI Jr. et al., 1994), the FIGS (Maxwell ME, 1992), and available medical records. Participants were recruited from bipolar research clinics, mental health organizations, families participating in alternate bipolar research projects, electronic and printed media, and public notice boards. |
| UMCU – BD twins | All twins were raised together, except for one control pair where twins were separated at 12 years of age when both parents died. Subjects were between 18 and 60 years of age at the time of enrolment in the study. Clinical diagnosis of Axis I psychiatric disorders and Axis II personality disorders was confirmed using the SCID and SIDP, respectively, and through available medical records. Patients were also interviewed on their medication history. The twin pairs had no history of drug or alcohol dependency for the last 6 months prior to inclusion in the study, for this was an exclusion criterion. Moreover, none had severe medical illness, verified with a medical history inventory. The current mood state of BD patients was assessed using the YMRS and the IDS. Upon inclusion, all patients were euthymic with a YMRS score of 4 or less and an IDS score of 12 or less, except for nine BD patients who were mildly to severely depressed or hypomanic. Healthy control pairs were matched to the bipolar pairs for zygosity, gender, age and parental education. Control pairs had no history of severe medical illness and had no first-degree relative with a history of a major Axis I psychiatric disorder (DSM-IV). Family histories of all twins were obtained via the Family Interview Genetic Studies, performed with both twins of each pair. Zygosity was determined with DNA fingerprinting using high polymorphic microsatellite markers 9 to 11. The medical ethics review board of the University Medical Center Utrecht approved the study and all participants gave written informed consent after full explanation of the study aims and procedures. (Van der Schot et al. 2009, Bootsman et al. 2015) |
| UMCU – DBSOS | This study includes participants between 8 and 18 years of age, including offspring of a patient with schizophrenia, offspring of a patient with bipolar disorder, and community control subjects. None met DSM-V criteria for schizophrenia or a related psychotic disorder at the time of baseline assessment (present and lifetime). For each family, all offspring in the appropriate age range entered our study to prevent a biased selection of participants within the family, as offspring with (subthreshold) symptoms may otherwise be more likely to be signed up for study participation than offspring with no (subthreshold) symptoms. Clinical diagnoses of parents were confirmed using the SCID-I. Control parents were screened for psychopathology using the mini-SCAN. The medical ethics committee of the University Medical Center Utrecht approved the study, and all participating children and their parents provided written informed consent. The K-SADS-PL was used to evaluate symptoms and DSM-V diagnoses of all participants. The majority of the offspring were naïve to psychotropic medication. (Collin et al. 2017) |
| UMCU – GROUP | Patients had to fulfil the following criteria: (1) age between 16 and 50 years, (2) meeting DSM-IV criteria for a nonaffective psychotic disorder (including schizophrenia, schizophreniform disorder, and schizoaffective disorder), (3) fluent in Dutch, and (4) able and willing to give written informed consent. Eligible siblings had to fulfil the criteria of (1) age between 16 and 50 years, (2) fluent in Dutch, and (3) able and willing to give written informed consent. Eligible healthy control subjects had to fulfil the criteria of (1) age between 16 and 50 years, (2) no lifetime psychotic disorder and/or use of lithium medication (in the past), (3) no first- or second-degree family member with a lifetime psychotic disorder, (4) fluent in Dutch, and (5) able and willing to give written informed consent. Presence or absence of psychopathology was established by using the CASH. Diagnosis was based on the DSM-IV criteria. Of all subjects, urine was screened for cocaine, amphetamines, and for cannabis. Subjects with substance dependence/abuse (based on the criteria of the CIDI [sections B, J, and L]) and a major medical or neurological illness were excluded. (Boos et al. 2012) |
| UMCU – Parents | Both parents of patients with schizophrenia were recruited at the University Medical Center Utrecht, as well as healthy control couples. The CASH, SADS-L, SIDP-IV, and the FIGS were obtained from all participants. Psychiatric diagnosis was established according to DSM-IV criteria. At least one of the children of the parents met DSM-IV criteria for schizophrenia on the basis of the CASH. Parents of patients were excluded if they had a history of psychotic illness. For control couples, exclusion followed in case of any axis-I DSM-IV diagnosis, or diagnosis of depression, manic depression, or psychotic disorder in first-degree family, or psychotic disorder in second-degree family. In both groups all participants were physically healthy and had no history of neurological illness, or drug or alcohol abuse. (Boos et al. 2011) |
| UMCU – UTWINS | 1.5T: Twin pairs discordant for schizophrenia, and healthy control twins were pairwise matched on zygosity, sex, age, and birth order took part in the study. Subjects were recruited in collaboration with psychiatric services and by advertisements in national newspapers. All subjects gave written informed consent to participate in the study. Zygosity was determined by DNA fingerprinting. Except for 1 control twin pair, all twins were reared together. The 1 control twin pair was separated at age 12 years when both their parents died. All subjects underwent extensive psychiatric assessment procedures using the CASH interview, the SADS-L, the Structured Interviews for DSM-III-R and DSM-IV, the FIGS, and a medical history inventory. Psychiatric diagnosis was established according to criteria of DSM- IV. The following subtypes were diagnosed in the twins with schizophrenia: paranoid, disorganized, undifferentiated, residual, and catatonic. Diagnoses in non-schizophrenic co-twins included paranoid personality disorder, schizotypal personality disorder, schizoid personality disorder, major depressive disorder, avoidant personality disorder, generalized anxiety disorder with a dependent personality disorder, and no psychiatric diagnoses. Moreover, some patients and co-twins had histories of substance or alcohol abuse. Healthy control twins had no schizophrenic spectrum disorders, no first-degree relatives with a history of psychiatric illness, and no second-degree relatives with a psychotic disorder. Two patients had never been on antipsychotic medication. (Baaré et al. 2001). 3T: U-TWIN consists of twins with discordance for schizophrenia and control twins. The control twins were selected to match the discordant twins on age, handedness, and parental educational level. There were more males in the discordant twin group compared with the control twins, which was corrected for statistically. Control twins were excluded if they ever met criteria for a psychotic or manic disorder or substance dependence, had a first-degree relative with schizophrenia, or were diagnosed as having a neurologic disorder. Zygosity of all twins was determined through testing polygenic genetic markers. The zygosity of incomplete pairs was known from participation in earlier studies. All subjects underwent psychiatric assessment by means of the CASH interview, symptom severity in the patients was assessed using the PANSS. Diagnoses were established using DSM-IV criteria. All but one patient received antipsychotic medication. The twins were recruited through the UMC Utrecht twin database, the participant database of the GROUP cohort, 3 national newspaper advertisement and local psychiatry clinics. All subjects from the previous cohort agreed to participate again in this new 3T MRI study; no data from previous measurements was used. The Medical Ethical Committee of the University Medical Center Utrecht approved this study, and the experiments were in accordance with the Declaration of Helsinki. All participants gave their written informed consent. The subject overlap with our previous twin cohort is 30.5% (and in case of overlap, only the 3T measurement was included). (Bohlken et al. 2016) |
| UNIBA | Participants included patients with schizophrenia, unaffected siblings and healthy subjects. All individuals were white Caucasian, from the Apulia region, and they were aged 18 to 65 years. The eligibility criteria for all participants were (a) no history of head trauma or loss of consciousness; (b) no current or lifetime history of medical or neurological disorders; (c) no lifetime history of substance use disorder; (d) no MRI contraindications (e.g. metal implants). Patients were required to fulfil diagnostic DSM-IV criteria for Schizophrenia, while unaffected relatives of patients and healthy volunteers were included if they had no lifetime history of psychiatric disorders. |

**Supplementary Table 2.** IQ test battery description, NA = not applicable

| Sample | Inclusion criteria |
| --- | --- |
| BPO_FLB | The neuropsychological battery includes the Wechsler Abbreviated Scale of Intelligence (WASI; four subtests: vocabulary, similarities, block design, and matrix reasoning), the Test of Nonverbal Intelligence (TONI), the Delis-Kaplan Executive Function Scale (D–KEFS; nine subtests: trail making, verbal fluency, design fluency, color-word interference (Stroop), sorting, twenty questions, word context, tower and proverb tests), the California Verbal Learning Test (CVLT), Benton line orientation, grooved pegboard, selected subtests from the Wechsler Adult Scale of Intelligence (WAIS-III) or Wechsler Intelligence Scale for Children (WISC-IV) depending on the age of the subject (digit span, and digit-symbol subtests), and the abbreviated Wechsler Individual Achievement Test (WIAT–II-A; three subtests: spelling, word reading, and numerical operations). In addition, computerized measures of attention (Continued Performance Test (CPT; (212))) and inhibition (Go-No (213)), as well as facial emotion identification (211) will be collected. |
| C_SFS | NA |
| Cardiff | NA |
| CLiNG – BD | NA |
| CLiNG – SZ | NA |
| DEU | NA |
| EGEU | NA |
| EHRS | Total IQ was measured by using the verbal (Information, Comprehension, Arithmetic, Digit Span, Similarities, and Vocabulary) and performance (Picture Arrangement, Picture Completion, Block Design, Object Assembly, and Digit Symbol) subtests of the Wechsler Adult Intelligence Scale—Revised (WAIS-R) |
| ENBD_UT | The neuropsychological battery includes the Wechsler Abbreviated Scale of Intelligence (WASI; four subtests: vocabulary, similarities, block design, and matrix reasoning) |
| FIDMAG-Clinic | Total IQ was measured using four subtests of the Wechsler Adult Intelligence Scale III (WAIS‐III) (vocabulary, similarities, block design, and matrix reasoning) |
| Geneva | NA |
| HUBIN | WAIS Vocabulary was used as a proxy for IQ |
| IDIBAPS | Intelligence quotient (IQ) was assessed using the Spanish version of the Wechsler Intelligence Scale for Children-Fourth Edition (WISC-IV) (Wechsler, 2003) which evaluates intellectual abilities in children and adolescents aged between 6 and 16 years old. The General Ability Index (GAI), derived from the VCI and PRI, was used as an index of intelligence level (Flanagan and Kaufman, 2008). |
| IoP – BD | NA |
| IoP – SZ | Current IQ was assessed with the Wechsler Adult Intelligence Scale–Third Edition |
| LIBD | The WAIS-R short-form full-scale IQ was used for estimating IQ |
| Maastricht – GROUP | For estimating the IQ the Wechsler Adult Intelligence Scale (WAIS; including subscales symbol, calculation, block design and information) was used. Based on the WAIS an estimated total IQ was be provided. |
| MFS | Total IQ was measured using a five sub-tests of the short form (vocabulary, similarities, comprehension, block design, object assembly)  of the Wechsler Adult Intelligence Scale–Revised (WAIS–R) |
| MooDS – BD | Total IQ was meaured using the MWT B (Mehrfach Wortschatz Test), a multiple choice vocabulary test that roughly measures verbal cristalline intelligence. Test results were translated to IQ values and mean corrected. |
| MooDS – SZ | otal IQ was meaured using the MWT B (Mehrfach Wortschatz Test), a multiple choice vocabulary test that roughly measures verbal cristalline intelligence. Test results were translated to IQ values and mean corrected. |
| MSSM | NA |
| Olin | Total IQ was estimated based on vocabulary and matrix reasoning from the Wechsler Abbreviated Scale of Intelligence (WASI, Wechsler, 1999). |
| ORBIS I | NA |
| ORBIS II | NA |
| PENS | Total IQ was estimated using the Vocabulary and Block Design subtests from the WAIS-III. |
| PHCP | Total IQ was estimated using the Similarities and Matrix Reasoning subtests from the WAIS-IV. |
| STAR (Swedish) BD twin cohort | NA |
| STAR (Swedish) SZ twin cohort | NA |
| SydneyBipolarGroup | For the majority of participants total IQ was estimated based on vocabulary and matrix reasoning from the Wechsler Abbreviated Scale of Intelligence (WASI, Wechsler, 1999). A small number of participants did not have a usable MRI scan at the first testing time point (baseline) so we used their data from the next timepoint that involved an MRI scan (follow-up 2). Whilst participants completed a WASI IQ test at baseline, participants at follow-up 2 completed the Wechsler Test of Adult Reading (WTAR) instead. |
| UMCU – BD twins | Four subtests of the Dutch version of the WAIS were used as a proxy for IQ, i.e. vocabulary, block design, picture arrangement and comprehension. The four subtests were used to calculate a proxy measure for the full-scale IQ. |
| UMCU – DBSOS | The total IQ score for each study group was estimated based on the performance of four subtests, Picture Arrangement, Block Design, Vocabulary and Information, of the Dutch version of the WAIS III in participants older than 16 years old4, or the Dutch version of the Wechsler Intelligence Scale for Children-Revised Wechsler Intelligence Scale for Children (WISC) III in the case of younger offspring. |
| UMCU – GROUP | The IQ scores were based on four subtests of the Dutch version of the WAIS III, digit-symbol coding, information, arithmetic, and block design4. The four subtests were used to calculate a proxy measure for the full-scale IQ. |
| UMCU – Parents | Current IQ was estimated using a short form of the Groningen Intelligence Test. |
| UMCU – UTWINS | Cohort I: An evaluation of intellectual ability was obtained by a shortened version of the Wechsler Adult Intelligence Scale (WAIS) III general intelligence test, consisting of five subtests: Digit Symbol-Coding, Block Design, Arithmetic, Digit Span, and Information. The five subtests were used to calculate a proxy measure for the full-scale IQ. Cohort II: Four subtests of the Dutch version of the WAIS were used as a proxy for IQ, i.e. vocabulary, block design, picture arrangement and comprehension. The four subtests were used to calculate a proxy measure for the full-scale IQ. |
| UNIBA | We estimated IQ using the Wechsler Adult Intelligence Scale-Revised. Furthermore, the Italian version of the Wide Reading Achievement Test was administered to obtain a measure of premorbid IQ for each participant. |

**Supplementary Table 3.** Educational attainment (i.e. years of education completed) criteria description, NA = not applicable

| Sample | Inclusion criteria |
| --- | --- |
| BPO_FLB | Years of Education Completed |
| C_SFS | Years of Education Completed |
| Cardiff | NA |
| CLiNG – BD | What is your highest finished secondary school qualification? 0) No education finished, 1) Lower Secondary education, 2) O-Level, 3) Higher school certificate (A level)  What is your highest professional qualification?  0) No professional qualification, 1) Vocational education, 2) University, 3) Doctorate This was converted to years of education |
| CLiNG – SZ | What is your highest finished secondary school qualification?  0) No education finished, 1) Lower Secondary education, 2) O-Level, 3) Higher school certificate (A level)  What is your highest professional qualification?  0) No professional qualification, 1) Vocational education, 2) University, 3) Doctorate This was converted to years of education |
| DEU | Years of Education Completed |
| EGEU | Years of Education Completed |
| EHRS | Age individuals left school, which was used to calculate years of education completed (age left school minus 5; based on the average age that children start school in Scotland) |
| ENBD_UT | Years of Education Completed |
| FIDMAG-Clinic | NA |
| Geneva | NA |
| HUBIN | Years of Education Completed |
| IDIBAPS | NA |
| IoP – BD | Demographic information including years of education was collected using a standardised interview. Both total years of completed education and highest completed academic qualification were recorded. |
| IoP – SZ | Demographic information including years of education was collected using a standardised interview. Both total years of completed education and highest completed academic qualification were recorded. |
| LIBD | Years of Education Completed |
| Maastricht – GROUP | NA |
| MFS | Education was measured by asking individuals the number of years they have been in education as well as highest completed academic qualification. |
| MooDS – BD | Education was measured by asking individuals the number of years they have been in education. |
| MooDS – SZ | Education was measured by asking individuals the number of years they have been in education. |
| MSSM | NA |
| Olin | Education was measured by asking individuals the number of years they have been in education, regardless of graduation |
| ORBIS I | NA |
| ORBIS II | NA |
| PENS | We ask for the subject’s highest completed grade or degree in years (with 12 being completion of High School or Equivalent). |
| PHCP | We ask for the subject’s highest completed grade or degree in years (with 12 being completion of High School or Equivalent). |
| STAR (Swedish) BD twin cohort | Highest Education?  1 = Elementary school (9 years in school) 2 = Realskola (similar to elementary school between the years 1905 to 1962 – the older twins attended this school) -> 9 years (approximately) 3 = High school 2 years 4 = High school 3 years 5 = University  6 = Other 7 = Don't know 8 = Do not want to answer |
| STAR (Swedish) SZ twin cohort | Highest Education?  1 = Elementary school (9 years in school) 2 = Realskola (similar to elementary school between the years 1905 to 1962 – the older twins attended this school) -> 9 years (approximately) 3 = High school 2 years 4 = High school 3 years 5 = University  6 = Other 7 = Don't know 8 = Do not want to answer |
| SydneyBipolarGroup | Years of Education Completed |
| UMCU – BD twins | What is your highest finished education with a diploma? 0) No education finished 1) Primary school only 2) Lower vocational education (LB0) 3) General secondary education (LAVO, MAVO) 4) Higher secondary education (HAVO) 5) Higher secondary education (VWO) 6) Intermediate vocational education (MBO) 7) Higher vocational education (HBO) 8) University This was converted to years of educational completed |
| UMCU – DBSOS | NA |
| UMCU – GROUP | What is your highest finished education with a diploma? 0) No education finished 1) Primary school only 2) Lower vocational education (LB0) 3) General secondary education (LAVO, MAVO) 4) Higher secondary education (HAVO) 5) Higher secondary education (VWO) 6) Intermediate vocational education (MBO) 7) Higher vocational education (HBO) 8) University This was converted to years of educational completed |
| UMCU – Parents | What is your highest finished education with a diploma? 0) No education finished 1) Primary school only 2) Lower vocational education (LB0) 3) General secondary education (LAVO, MAVO) 4) Higher secondary education (HAVO) 5) Higher secondary education (VWO) 6) Intermediate vocational education (MBO) 7) Higher vocational education (HBO) 8) University This was converted to years of educational completed |
| UMCU – UTWINS | What is your highest finished education with a diploma? 0) No education finished 1) Primary school only 2) Lower vocational education (LB0) 3) General secondary education (LAVO, MAVO) 4) Higher secondary education (HAVO) 5) Higher secondary education (VWO) 6) Intermediate vocational education (MBO) 7) Higher vocational education (HBO) 8) University This was converted to years of educational completed |
| UNIBA | Years of Education Completed, including graduate school and medical specialization |

**Supplementary Table 4.** Sample image acquisition and image processing details

| Sample | Number of Scanners | Scanner Vendor & Type | Imaging Protocols | Slice Orientation | FreeSurfer Version | Operating System/Linux Kernel Version |
| --- | --- | --- | --- | --- | --- | --- |
| BPO_FLB | 1 | 3.0T Siemens Allegra | T1-weighted scans were acquired using a three-dimensional magnetization prepared rapid gradient echo (3DMPRAGE) protocol with the following parameters. Repetition time (TR) = 1750 ms, echo time (TE) = 4.38 ms, flip angle = 8°, Slice thickness = 1mm, matrix size = 256 x 208 and voxel size = 1 mm. |  | v5.3.0 |  |
| C_SFS | 1 | 3T General Electric Discovery MR750 | Each scan consisted of a whole-brain T1-weighted 3D magnetization-prepared rapid gradient-echo (MP-RAGE) sequence with the following parameters: echo time (TE)=3.1ms, inversion time (TI)=650ms, repetition time (TR)=7.4ms, flip angle=11°, field of view (FOV)=25.6, matrix=256×256, slice thickness=1mm, 236 coronal slices. |  | v6.0.0 |  |
| Cardiff | 1 | GE HDx 3T scanner | T1 - axial 3D fast spoiled gradient recalled (FSPGR) sequence (TR/TE/TI = 8/3/ 450 ms; Flip Angle = 200; acquisition matrix= 256(AP)x192(LR)x172(SI), 1mm isotropic voxels) |  | v5.3.0 | 3.0.80-0.7-default |
| CLiNG | 1 | 3T Magnetom TIM Trio | MRI scanning was performed on a 3.0-Tesla Magnetom TIM Trio (Siemens, Erlangen, Germany). A T1-weighted, 3D magnetization prepared rapid gradient echo sequence (MPRAGE) (TR/TE/TI/FA=2250 ms/3.26 ms/900 ms/9°; image matrix = 256 x 256; duration 8 min and 26 sec) was acquired generating 192 sagittal slices with a voxel size of 1 mm3. | Sagittal | v5.3.0 | Ubuntu 12.04: 2.6.32-431.17.1.el6.x86_64 |
| DEU | 1 | 1.5 T Philips Tesla Achieva MRI | 3D T1-fast field echo (FFE) axial images were acquired with the following parameters: repetition time (TR) =8.7 ms, echo time (TE) =4 ms, flip angle=8 o, field of view (FOV) =230 mm x 220 mm, slice thickness=1 mm, number of signal averages (NSA) =1, matrix=192 |  | v5.3.0 | 2.6.32-573.12.1.el6.x86_64 |
| EGEU | 1 | Siemens 3T Magnetom Verio | T1-weighted anatomical 3D (MP-RAGE) 1 mm3 isotropic (FoV=256, TR=1600 msec, TE=221 msec, TI= 900 msec, FA=9°), matrix 256X256 |  | v5.3.0 | 2.6.32-431.17.1.el6.x86_64 |
| EHRS | 1 | 1T Siemens | scanned with a 1 Tesla 42 SPE Siemens MRI scanner (Siemens, Erlangen, Germany). 128 contiguous coronal T1-weighted slices (thickness 1.88 mm, field-of-view 250 × 250 mm) were obtained using a Magnetisation Prepared Rapid Acquisition of Gradient Echo (MPRAGE)sequence (TR=10ms, TE=4ms, TI=200ms, relaxation time 500ms). | Coronal | v5.3.0 | Linux: 2.6.32-754.2.1.e16.x86_64 |
| ENBD_UT | 1 | Philips 3 Tesla | T1-weighted, axial, 25.6cm x 25.6 cm square field-of-view (1.0mm slice, Tr=1750msec, Te=4.4msec, Ti=900msec, flip=80, data acquisition matrix=256(phase)x256(frequency)x(160 slice). |  | v5.3.0 |  |
| FIDMAG-Clinic | 1 | 1.5 Tesla GE Signa | T1‐weighted MRI data were acquired using 180 contiguous slices with thickness of 1 mm. The images were collected in a 256 × 224 acquisition matrix and were zero‐filled in the k‐space by the scanner to yield an image of 512 × 512 pixels with reconstruction diameter of 240 mm, resulting in an effective in‐plane voxel size of 0.47 × 0.47 mm2. The echo (TE), repetition (TR) and inversion (TI) times were equal to (TE/TR/TI) = 3.93 ms/2000 ms/710 ms respectively. The flip angle was 15 degrees. | Axial | v6.0.0 | Ubuntu 18.04 x86_64 |
| Geneva | 1 | 3T Siemens Trio | 32 channels head-coil, 3D T1-weighted images, 192 sagittal slices, TR 1900 ms, TE: 2.27 ms, Voxel size: 1.0×1.0×1.0 mm, 9° flip angle, Field of view 256 mm, Acquisition Matrix 256x256 mm | sagittal | v6.0.0 | Ubuntu 16.04 |
| HUBIN | 1 | 1.5T GE Signa | 3D spoiled gradient recalled pulse sequence for T1-weighted images: 1.5 mm coronal slices, no gap, 35° flip angle, repetition time 24 ms, echo time 6.0 ms, number of excitations 2, field of view 24 cm, acquisition matrix 256 × 192. |  | v5.3.0 | 3.13.0-79-generic |
| IDIBAPS | 1 | Siemens Trio 3T | 240 sagittal slices, 2,300-ms repetition time, 3.01-ms echo time, 1-mm slice thickness, 900-ms inversion time, 394x240 field of view, 256x256 matrix size, and 9 degrees flip angle. |  | v5.3.0 | 2.6.32.12-0.7; 3.0.76-0.11 |
| IoP – BD | 1 | 1.5 Tesla GE N/Vi Signa System scanner | Coronal FSPGR. Matrix: 256 X 256, 124 slices with 1.5mm slice thickness. FOV: 220x160. Flip angle: 20°. Number of excitations: 1. No gap. RT=13.1ms echo time = 5.8ms TI=450ms. Matched to MFS. |  | v5.3.0 | 2.6.32-358.6.2.el6.x86_64 |
| IoP – SZ | 1 | 1.5 Tesla GE N/Vi Signa System scanner | Participants underwent MRI scanning on a General Electric Signa Advantage scanner at 1.5 Tesla. A 3-dimensional T1-weighted, coronal, spoiled gradient (SPGR) of the whole head was obtained (TE=5ms, TR=35ms, flip angle=30°, NEX=1, FOV=200x200mm, voxel dimensions=1x1x1.5mm), yielding 124 contiguous slices 1.5mm thick. Imaging took place on identical scanners with identical protocols at either of two sites (St Georges Hospital, London, or The Maudsley Hospital, London) |  | v5.3.0 | 2.6.32-358.6.2.el6.x86_64 |
| LIBD | 1 | 1.5T GE | T1-weighted spoiled gradient recalled sequence (spgr). Repetition time, 24 milliseconds; echo time, 5 milliseconds; number of excitations, 1; flip angle, 45 degrees; matrix size 256 x 256; field of view, 24 x 24 cm; 124 sagittal slices (0.94 x 0.94 x 1.5 mm). | Sagittal | v5.0.0 | Linux: 2.6.32-696.23.1.e16.x86_64 |
| Maastricht – GROUP | 1 | 3T Siemens Magnetom Allegra | Modified Driven Equilibrium Fourier Transform sequence (MDEFT); TR=7.92msec, TE=2.4msec, IR=910msec, flip angle=15°, FOV=256x240. Acquisition Matrix=256x240x176 (1 x 1 x 1mm). Magnetisation Prepared Rapid Acquisition of Gradient Echo (MPRAGE); TR=2250msec, TE=2.6msec, IR=900msec, flip angle=9°, FOV=256x256. Acquisition Matrix=256x256x192 (1 x 1 x 1mm). | Sagittal | v5.3.0 | macOS: 10.8.0 |
| MFS | 1 | 1.5T GE N/Vi Signa System | 3D T1-weighted spoiled gradient recall echo sequence (SPGR). TR=13.1 ms, TI=450 ms, TE=5.8 ms, number of excitations=1, flip angle=20°, acquisition matrix=256X256X128, 1.5mm thick contiguous coronal slices. |  | v5.3.0 | 3.0.0-21-generic |
| MooDS | 1 | Siemens Trio 3T | T1-weighted anatomical 3D (MP-RAGE) 1 mm3 isotropic (FoV=192, TR=1.57 s, TE=2.74 ms, FA=15°) |  | v5.3.0 | 4.4.0-142-generic |
| MSSM | 1 | 1.5T GE Signa | 3D T1-weighted spoiled gradient recalled acquisition in steady state; Voxel Size: 0.9375x0.9375x1.5mm3, TR/TE/TI=5.1/18/450 ms, Flip Angle: 20°. | Axial | v5.3.0 | 2.6.32-358.6.2.el6.x86_64 |
| Olin | 1 | Siemens Magnetom Allegra 3T | 3D magnetization-prepared rapid gradient-echo (MPRage) sequence: TI=766; TR=2200; TE=4.13; flip angle 13 deg; FOV 256 mm; 0.8mm iso; axial slices parallel to the AC-PC line. To increase signal-to-noise ratio, four volumes were acquired per subject. |  | v5.3.0 | Linux : 2.6.32-504.16.2.el6.x86_64 |
| ORBIS I | 1 | 1.5-Tesla GE Signa | We acquired T1-weighted SPGR (Spoiled Gradient Recalled) scans: flip angle=40°, TE=5 ms, TR=25 ms, FOV=24 cm x18 cm, matrix=256x160 pixels, NEX=1, no inter-slice gap, 124 coronal, 1.5 mm thick slices. |  | v5.3.0 | macOS |
| ORBIS II | 1 | 1.5-Tesla GE Signa | We acquired T1-weighted SPGR (Spoiled Gradient Recalled) scans: flip angle=40°, TE=5 ms, TR=25 ms, FOV=24 cm x18 cm, matrix=256x160 pixels, NEX=1, no inter-slice gap, 124 coronal, 1.5 mm thick slices. |  | v5.3.0 | macOS |
| PENS | 1 | 3T Siemens Trio | 32 channels head-coil, 3D T1-weighted MP-RAGE images, 239 sagittal slices, TR 2400 ms, TE: 2.12 ms, Voxel size: 1.0×1.0×1.0 mm, 8° flip angle, Inversion time 1060 ms, Field of view 256 x 240 mm, Acquisition Matrix 256 x 240 | double oblique Sagittal | v5.3.0 | Linux 2.6.32-74-generic x86_64 |
| PHPC | 1 | 3T Siemens Prisma | 32 channels head-coil, 3D T1-weighted multi-echo MP-RAGE images, 208 double oblique sagittal slices, TR 2500 ms, TE: 1.81/3.6/5.39/7.18 ms, Voxel size: 0.8×0.8×0.8 mm, 8° flip angle, Inversion time 1000 ms, Field of view 256 x 240 mm, Acquisition Matrix 320 x300 | double oblique Sagittal | v5.3.0 | Linux cn0456 3.10.0-957.27.2.e17.x68_64 |
| STAR (Swedish) BD twin cohort | 1 | GE 1.5T Signa |  |  | v5.3.0 | Linux: 3.10.0-693.43.1.el7.x86_64 |
| STAR (Swedish) SZ twin cohort | 1 | GE 1.5T Signa | T1 - sagittal irSPGR sequence, 1mm3 isotropic voxels, 256mm FOV, TR/TE = 25/6 msec, 35 degree flip |  | v5.3.0 | Linux: 3.10.0-693.43.1.el7.x86_64 |
| SydneyBipolarGroup | 1 | Philips Achieva 3T | 180 T1-weighted 3D turbo field-echo images were acquired sagitally (TR=5.5msec, TE=2.5ms, flip angle=8°, field of view=256x256x180mm, voxel size=1x1x1mm, scan time=371s). | Sagittal | v5.3.0 | Linux : 2.6.32-504.3.3.el6.x86_64 |
| UMCU – BD twins | 1 | 1.5T Philips NT | The acquired scans were T1-weighted, 3-dimensional, fast-field echo scans with 160-180 contiguous coronal slices (256×256 matrix, echo time = 4.6ms, repetition time = 30ms, flip angle = 30°, 1×1×1.2 mm3 voxels, field of view = 256mm/70%). |  | v5.3.0 | 2.6.32-358.6.2.el6.x86_64 |
| UMCU – DBSOS | 1 | 3T Philips Achieva | The T1-weighted 3-dimensional fast-field echo scans were acquired with the following parameters: 220 0.8 mm contiguous slices, echo time = 4.6 ms, repetition time = 10 ms, flip angle = 8°, in-plane voxel size 0.75x0.75 mm². |  | v5.3.0 | 2.6.32-358.6.2.el6.x86_64 |
| UMCU – GROUP | 1 | 1.5T Philips Achieva | The acquired scans were T1-weighted, 3-dimensional, fast-field echo scans with 160-180 contiguous coronal slices (256×256 matrix, echo time = 4.6ms, repetition time = 30ms, flip angle = 30°, 1×1×1.2 mm3 voxels, field of view = 256mm/70%). |  | v5.1.0 | 2.6.32-358.6.2.el6.x86_64 |
| UMCU – Parents | 1 | 1.5T Philips NT | The acquired scans were T1-weighted, 3-dimensional, fast-field echo scans with 160-180 contiguous coronal slices (256×256 matrix, echo time = 4.6ms, repetition time = 30ms, flip angle = 30°, 1×1×1.2 mm3 voxels, field of view = 256mm/70%). |  | v5.3.0 | 2.6.32-358.6.2.el6.x86_64 |
| UMCU – UTWINS | 2 | 1.5T Philips NT/3T Philips Achieva | 1.5T: The acquired scans were T1-weighted, 3-dimensional, fast-field echo scans with 160-180 contiguous coronal slices (256×256 matrix, echo time = 4.6ms, repetition time = 30ms, flip angle = 30°, 1×1×1.2 mm3 voxels, field of view = 256mm/70%). 3T: The T1-weighted 3-dimensional fast-field echo scans were acquired with the following parameters: 220 0.8 mm contiguous slices, echo time = 4.6 ms, repetition time = 10 ms, flip angle = 8°, in-plane voxel size 0.75x0.75 mm². |  | v5.3.0 | 2.6.32-358.6.2.el6.x86_64 |
| UNIBA | 1 | GE 3T | 124 1.3-mm slices using 3D T1-weighted gradient echo fast SPGR sequence (TE=min full; flip angle, 6°; prep time, 725; field of view, 250 mm; bandwidth, 31.25; matrix, 256 x 256) |  | v5.3.0 | 4.4.0-116-generic |

**Supplementary Table 5.** Cohen’s d effect size bipolar and schizophrenia relatives, controlled for IQ (middle column) and controlled for educational attainment (right column)

|  |  | BIPOLAR DISORDER RELATIVE | | | SCHIZOPHRENIA RELATIVE | | |
| --- | --- | --- | --- | --- | --- | --- | --- |
|  |  | ES ± 95% CI | IQ  ES ± 95% CI | EA  ES ± 95% CI | ES ± 95% CI | IQ | EA |
|  | *Global measures* |  |  |  |  |  |  |
|  |  |  |  |  |  |  |  |
|  | ICV | 0.17 [0.06 0.28]** | 0.21 [0.05 0.36]* | 0.25 [0.11 0.4]** | -0.03 [-0.13 0.06] | 0.05 [-0.03 0.13] | 0.06 [-0.04 0.16] |
|  | Surface area | 0.12 [-0.00 0.24] | 0.12 [-0.05 0.29] | 0.19 [0.04 0.34]* | -0.02 [-0.13 0.09] | 0.04 [-0.05 0.13] | 0.09 [-0.03 0.21] |
|  | Cortical thickness | -0.05 [-0.15 0.06] | -0.06 [-0.19 0.07] | -0.06 [-0.26 0.13] | -0.10 [-0.23 0.03] | -0.15 [-0.29 -0.01]* | -0.1 [-0.24 0.04] |
|  |  |  |  |  |  |  |  |
|  |  |  |  |  |  |  |  |
|  | Total brain | 0.08 [-0.04 0.2] | 0.13 [-0.03 0.3] | 0.15 [-0 0.29] | -0.12 [-0.22 -0.02]* | -0.03 [-0.11 0.05] | 0 [-0.12 0.11] |
|  | Cortical GM | 0.1 [-0.03 0.23] | 0.13 [-0.06 0.33] | 0.18 [0.01 0.36]* | -0.09 [-0.21 0.03] | -0.03 [-0.12 0.07] | 0.05 [-0.05 0.15] |
|  | Cerebral WM | 0.06 [-0.04 0.17] | 0.13 [-0.02 0.28] | 0.1 [-0.05 0.25] | -0.11 [-0.19 -0.02]* | -0.01 [-0.09 0.07] | -0.03 [-0.13 0.08] |
|  | Cerebellum GM† | 0.07 [-0.09 0.22] | 0.02 [-0.16 0.2] | 0.09 [-0.09 0.27] | -0.12 [-0.2 -0.04]** | -0.05 [-0.13 0.03] | -0.02 [-0.15 0.1] |
|  | Cerebellum WM† | -0.03 [-0.17 0.11] | -0.06 [-0.23 0.11] | 0.05 [-0.13 0.23] | -0.1 [-0.18 -0.01]* | -0.03 [-0.11 0.05] | -0.02 [-0.16 0.11] |
|  | Third ventricle | 0 [-0.11 0.1] | -0.1 [-0.25 0.04] | -0.05 [-0.19 0.09] | 0.13 [0.01 0.25]* | 0.15 [0 0.29]* | 0.15 [0 0.31]* |
|  | Lateral ventricles | 0.12 [0.04 0.21]** | 0.12 [-0.02 0.27] | 0.05 [-0.09 0.19] | 0.05 [-0.03 0.13] | 0.12 [0.03 0.21]* | 0.08 [-0.02 0.19] |
|  |  |  |  |  |  |  |  |
|  | *Subcortical volumes* |  |  |  |  |  |  |
|  |  |  |  |  |  |  |  |
|  | Thalamus | 0.02 [-0.09 0.13] | 0.14 [0.02 0.26]* | 0.02 [-0.12 0.17] | -0.09 [-0.19 -0]* | -0.03 [-0.16 0.09] | 0.01 [-0.12 0.14] |
|  | Caudate | 0.18 [0.09 0.26]** | 0.23 [0.12 0.35]** | 0.11 [-0.03 0.26] | 0 [-0.1 0.1] | 0.03 [-0.06 0.13] | 0.06 [-0.05 0.16] |
|  | Putamen | 0.05 [-0.06 0.15] | 0.11 [-0.05 0.27] | 0.04 [-0.1 0.18] | -0.02 [-0.13 0.09] | 0.04 [-0.07 0.15] | 0.01 [-0.11 0.13] |
|  | Pallidum | 0.04 [-0.07 0.15] | 0.14 [-0.01 0.28] | 0.06 [-0.11 0.22] | 0.02 [-0.1 0.13] | 0.07 [-0.06 0.19] | 0.07 [-0.05 0.19] |
|  | Hippocampus | 0 [-0.1 0.11] | 0.02 [-0.1 0.13] | -0.01 [-0.16 0.13] | -0.11 [-0.19 -0.03]** | -0.05 [-0.14 0.03] | 0 [-0.12 0.11] |
|  | Amygdala | 0.01 [-0.08 0.1] | 0.06 [-0.06 0.19] | -0.01 [-0.16 0.13] | -0.04 [-0.13 0.06] | 0.02 [-0.08 0.12] | 0.08 [-0.02 0.18] |
|  | Accumbens | 0.08 [-0.05 0.2] | 0.19 [0.01 0.37]* | 0.02 [-0.13 0.16] | -0.08 [-0.19 0.02] | -0.08 [-0.17 0.01] | -0.04 [-0.17 0.08] |
|  |  |  |  |  |  |  |  |

* p < 0.05, uncorrected | ** q < 0.05, corrected | † excluded Olin in cerebellum analyses

**Supplementary Table 6.** Cohen’s d effect size bipolar and schizophrenia patients, controlled for IQ (middle column) and controlled for educational attainment (right column)

|  |  | BIPOLAR DISORDER PATIENT^#^ | | | SCHIZOPHRENIA PATIENT | | |
| --- | --- | --- | --- | --- | --- | --- | --- |
|  |  | ES ± 95% CI | IQ  ES ± 95% CI | EA  ES ± 95% CI | ES ± 95% CI | IQ | EA |
|  | *Global measures* |  |  |  |  |  |  |
|  |  |  |  |  |  |  |  |
|  | ICV | 0.05 [-0.12 0.21] | 0.04 [-0.38 0.45] | 0.16 [-0.05 0.37] | -0.15 [-0.32 0.02] | 0.04 [-0.12 0.19] | -0.07 [-0.23 0.08] |
|  | Surface area | 0.02 [-0.17 0.21] | 0.03 [-0.24 0.29] | 0.08 [-0.20 0.36] | -0.14 [-0.34 0.07] | 0.03 [-0.15 0.21] | -0.16 [-0.34 0.02] |
|  | Cortical thickness | -0.31 [-0.55 -0.07]* | -0.28 [-0.52 -0.03]* | -0.31 [-0.57 -0.04]* | -0.52 [-0.74 -0.31]** | -0.56 [-0.86 -0.25]** | -0.54 [-0.74 -0.34]** |
|  |  |  |  |  |  |  |  |
|  |  |  |  |  |  |  |  |
|  | Total brain | -0.19 [-0.36 -0.01]* | -0.11 [-0.41 0.2] | -0.11 [-0.37 0.14] | -0.39 [-0.59 -0.2]** | -0.16 [-0.27 -0.05]** | -0.34 [-0.53 -0.15]** |
|  | Cortical GM | -0.13 [-0.33 0.06] | -0.08 [-0.37 0.22] | -0.05 [-0.26 0.16] | -0.45 [-0.65 -0.24]** | -0.24 [-0.39 -0.09]** | -0.41 [-0.62 -0.2]** |
|  | Cerebral WM | -0.16 [-0.32 -0]* | -0.03 [-0.3 0.24] | -0.1 [-0.38 0.19] | -0.29 [-0.46 -0.12]** | -0.05 [-0.16 0.06] | -0.23 [-0.4 -0.06]** |
|  | Cerebellum GM† | -0.17 [-0.34 -0.01]* | -0.08 [-0.28 0.12] | -0.14 [-0.37 0.09] | -0.27 [-0.42 -0.12]** | -0.11 [-0.3 0.07] | -0.14 [-0.26 -0.03]** |
|  | Cerebellum WM† | -0.06 [-0.21 0.08] | -0.14 [-0.31 0.04] | 0.02 [-0.2 0.25] | -0.2 [-0.36 -0.04]** | -0.05 [-0.2 0.09] | -0.11 [-0.25 0.04] |
|  | Third ventricle | 0.39 [0.18 0.6]** | 0.27 [-0.21 0.75] | 0.44 [0.09 0.79]* | 0.51 [0.38 0.64]** | 0.5 [0.28 0.72]** | 0.51 [0.37 0.65]** |
|  | Lateral ventricles | 0.39 [0.18 0.59]** | 0.35 [-0.07 0.77] | 0.44 [0.15 0.72]** | 0.34 [0.22 0.46]** | 0.42 [0.32 0.52]** | 0.37 [0.24 0.5]** |
|  |  |  |  |  |  |  |  |
|  | *Subcortical volumes* |  |  |  |  |  |  |
|  |  |  |  |  |  |  |  |
|  | Thalamus | -0.21 [-0.39 -0.03]* | -0.08 [-0.37 0.21] | -0.17 [-0.4 0.07] | -0.14 [-0.31 0.03] | 0.01 [-0.14 0.16] | -0.11 [-0.31 0.09] |
|  | Caudate | -0.02 [-0.15 0.11]* | -0.02 [-0.28 0.25] | 0.01 [-0.16 0.18] | 0.11 [-0.03 0.25] | 0.19 [0.09 0.28]** | 0.13 [-0.01 0.27] |
|  | Putamen | -0.1 [-0.33 0.13] | 0 [-0.41 0.42] | -0.1 [-0.4 0.2] | 0.16 [0.05 0.27]** | 0.21 [0.1 0.32]** | 0.19 [0.06 0.33]** |
|  | Pallidum | 0.05 [-0.12 0.22] | 0.07 [-0.2 0.35] | 0.05 [-0.13 0.22] | 0.28 [0.13 0.43]** | 0.35 [0.18 0.52]** | 0.32 [0.12 0.52]** |
|  | Hippocampus | -0.17 [-0.32 -0.01]* | -0.09 [-0.48 0.29] | -0.12 [-0.26 0.03] | -0.33 [-0.54 -0.12]** | -0.25 [-0.46 -0.04]** | -0.24 [-0.45 -0.03]** |
|  | Amygdala | -0.06 [-0.2 0.08] | 0.1 [-0.13 0.33] | -0.12 [-0.33 0.1] | -0.18 [-0.36 -0.01]* | -0.02 [-0.21 0.17] | -0.11 [-0.32 0.1] |
|  | Accumbens | -0.13 [-0.36 0.1] | -0.08 [-0.37 0.21] | -0.24 [-0.56 0.07] | -0.14 [-0.3 0.02] | -0.11 [-0.34 0.1] | -0.19 [-0.36 -0.02]** |
|  |  |  |  |  |  |  |  |

* p < 0.05, uncorrected | ** q < 0.05, corrected | † excluded Olin in cerebellum analyses | # lithium corrected

**Supplementary Table 7.** Cohen’s d effect size bipolar and schizophrenia relatives corrected for ICV (except ICV, SA and CT), controlled for IQ (middle column) and controlled for educational attainment (right column)

|  |  | BIPOLAR DISORDER RELATIVE | | | SCHIZOPHRENIA RELATIVE | | |
| --- | --- | --- | --- | --- | --- | --- | --- |
|  |  | ES ± 95% CI | IQ  ES ± 95% CI | EA  ES ± 95% CI | ES ± 95% CI | IQ | EA |
|  | *Global measures* |  |  |  |  |  |  |
|  |  |  |  |  |  |  |  |
|  | ICV | 0.17 [0.06 0.28]** | 0.21 [0.05 0.36]* | 0.25 [0.11 0.4]** | -0.03 [-0.13 0.06] | 0.05 [-0.03 0.13] | 0.06 [-0.04 0.16] |
|  | Surface area | 0.12 [-0.00 0.24] | 0.12 [-0.05 0.29] | 0.19 [0.04 0.34]* | -0.02 [-0.13 0.09] | 0.04 [-0.05 0.13] | 0.09 [-0.03 0.21] |
|  | Cortical thickness | -0.05 [-0.15 0.06] | -0.06 [-0.19 0.07] | -0.06 [-0.26 0.13] | -0.10 [-0.23 0.03] | -0.15 [-0.29 -0.01]* | -0.1 [-0.24 0.04] |
|  |  |  |  |  |  |  |  |
|  |  |  |  |  |  |  |  |
|  | Total brain | -0.10 [-0.22 0.03] | -0.06 [-0.22 0.09] | -0.08 [-0.23 0.07] | -0.16 [-0.24 -0.08]** | -0.14 [-0.22 -0.05]** | -0.13 [-0.28 0.01] |
|  | Cortical GM | -0.02 [-0.14 0.10] | -0.01 [-0.17 0.16] | 0.04 [-0.11 0.2] | -0.09 [-0.20 0.02] | -0.1 [-0.2 -0.00]* | -0.02 [-0.15 0.1] |
|  | Cerebral WM | -0.12 [-0.23 -0.00]* | -0.05 [-0.23 0.12] | -0.12 [-0.31 0.07] | -0.12 [-0.19 -0.04]** | -0.09 [-0.17 -0.01]* | -0.16 [-0.26 -0.05]** |
|  | Cerebellum GM† | -0.02 [-0.16 0.13] | -0.09 [-0.26 0.08] | -0.06 [-0.25 0.13] | -0.11 [-0.18 -0.03]** | -0.07 [-0.15 0.02] | -0.06 [-0.17 0.05]* |
|  | Cerebellum WM† | -0.14 [-0.27 -0.00]* | -0.2 [-0.38 -0.02]* | -0.08 [-0.27 0.11] | -0.09 [-0.17 -0.02]* | -0.06 [-0.14 0.02] | -0.08 [-0.21 0.05] |
|  | Third ventricle | -0.06 [-0.16 0.04] | -0.16 [-0.28 -0.04]* | -0.10 [-0.25 0.04] | 0.15 [0.04 0.27]** | 0.14 [-0.00 0.27] | 0.12 [-0.02 0.26] |
|  | Lateral ventricles | 0.07 [-0.04 0.17] | 0.06 [-0.09 0.21] | -0.03 [-0.17 0.11] | 0.09 [0.01 0.17]* | 0.14 [0.04 0.25]* | 0.06 [-0.04 0.17] |
|  |  |  |  |  |  |  |  |
|  | *Subcortical volumes* |  |  |  |  |  |  |
|  |  |  |  |  |  |  |  |
|  | Thalamus | -0.05 [-0.17 0.07] | 0.06 [-0.06 0.18] | -0.07 [-0.22 0.08] | -0.05 [-0.16 0.06] | -0.02 [-0.18 0.13] | 0.03 [-0.1 0.17] |
|  | Caudate | 0.13 [0.03 0.23]* | 0.18 [0.05 0.31]* | 0.08 [-0.06 0.22] | 0.03 [-0.06 0.12] | 0.03 [-0.07 0.14] | 0.06 [-0.06 0.18] |
|  | Putamen | 0 [-0.09 0.1] | 0.07 [-0.05 0.19] | 0.01 [-0.16 0.17] | 0.02 [-0.08 0.12] | 0.04 [-0.07 0.15] | 0.02 [-0.1 0.14] |
|  | Pallidum | -0.01 [-0.11 0.1] | 0.05 [-0.11 0.21] | 0.02 [-0.14 0.18] | 0.04 [-0.07 0.14] | 0.03 [-0.09 0.15] | 0.07 [-0.05 0.19] |
|  | Hippocampus | -0.04 [-0.17 0.09] | -0.05 [-0.21 0.1] | -0.08 [-0.25 0.1] | -0.09 [-0.16 -0.01]* | -0.07 [-0.16 0.02] | 0 [-0.12 0.11] |
|  | Amygdala | -0.03 [-0.12 0.06] | 0 [-0.11 0.12] | -0.05 [-0.19 0.09] | -0.01 [-0.09 0.08] | 0.01 [-0.12 0.13] | 0.09 [-0.01 0.2] |
|  | Accumbens | 0.05 [-0.07 0.17] | 0.14 [-0.05 0.32] | 0 [-0.14 0.14] | -0.06 [-0.14 0.02] | -0.1 [-0.2 0] | -0.04 [-0.17 0.1] |
|  |  |  |  |  |  |  |  |

* p < 0.05, uncorrected | ** q < 0.05, corrected | † excluded Olin in cerebellum analyses | # lithium corrected

**Supplementary Table 8.** Cohen’s d effect size bipolar and schizophrenia patients corrected for ICV (except ICV, SA and CT), controlled for IQ (middle column) and controlled for educational attainment (right column)

|  |  | BIPOLAR DISORDER PATIENT | | | SCHIZOPHRENIA PATIENT | | |
| --- | --- | --- | --- | --- | --- | --- | --- |
|  |  | ES ± 95% CI | IQ  ES ± 95% CI | EA  ES ± 95% CI | ES ± 95% CI | IQ | EA |
|  | *Global measures* |  |  |  |  |  |  |
|  |  |  |  |  |  |  |  |
|  | ICV | 0.05 [-0.12 0.21] | 0.04 [-0.38 0.45] | 0.16 [-0.05 0.37] | -0.15 [-0.32 0.02] | 0.04 [-0.12 0.19] | -0.07 [-0.23 0.08] |
|  | Surface area | 0.02 [-0.17 0.21] | 0.03 [-0.24 0.29] | 0.08 [-0.20 0.36] | -0.14 [-0.34 0.07] | 0.03 [-0.15 0.21] | -0.16 [-0.34 0.02] |
|  | Cortical thickness | -0.31 [-0.55 -0.07]** | -0.28 [-0.52 -0.03]* | -0.31 [-0.57 -0.04]* | -0.52 [-0.74 -0.31]** | -0.56 [-0.86 -0.25]** | -0.54 [-0.74 -0.34]** |
|  |  |  |  |  |  |  |  |
|  |  |  |  |  |  |  |  |
|  | Total brain | -0.41 [-0.58 -0.24]** | -0.22 [-0.53 0.10] | -0.41 [-0.69 -0.13]* | -0.49 [-0.69 -0.29]** | -0.37 [-0.46 -0.27]** | -0.49 [-0.77 -0.22]** |
|  | Cortical GM | -0.25 [-0.44 -0.06]** | -0.19 [-0.47 0.10] | -0.22 [-0.40 -0.04]* | -0.50 [-0.69 -0.31]** | -0.39 [-0.6 -0.18]** | -0.52 [-0.79 -0.26]** |
|  | Cerebral WM | -0.32 [-0.50 -0.13]** | -0.14 [-0.41 0.13] | -0.34 [-0.64 -0.04]* | -0.28 [-0.44 -0.11]** | -0.14 [-0.24 -0.05]** | -0.26 [-0.47 -0.05]** |
|  | Cerebellum GM† | -0.24 [-0.39 -0.10]** | -0.19 [-0.36 -0.01]* | -0.28 [-0.51 -0.04]* | -0.23 [-0.36 -0.10]** | -0.14 [-0.32 0.05] | -0.15 [-0.26 -0.04]** |
|  | Cerebellum WM† | -0.13 [-0.29 0.02] | -0.27 [-0.44 -0.09]* | -0.08 [-0.33 0.17] | -0.14 [-0.27 -0.02]** | -0.08 [-0.21 0.06] | -0.09 [-0.22 0.04] |
|  | Third ventricle | 0.36 [0.15 0.56]** | 0.28 [-0.16 0.73] | 0.36 [0.02 0.71]* | 0.57 [0.44 0.71]** | 0.53 [0.31 0.76]** | 0.55 [0.38 0.71]** |
|  | Lateral ventricles | 0.34 [0.12 0.56]** | 0.36 [-0.06 0.78] | 0.34 [0.02 0.66]* | 0.41 [0.27 0.55]** | 0.44 [0.31 0.57]** | 0.44 [0.28 0.59]** |
|  |  |  |  |  |  |  |  |
|  | *Subcortical volumes* |  |  |  |  |  |  |
|  |  |  |  |  |  |  |  |
|  | Thalamus | -0.3 [-0.51 -0.09]** | -0.11 [-0.31 0.09] | -0.33 [-0.63 -0.02]* | -0.07 [-0.20 0.06] | 0.01 [-0.15 0.18] | -0.07 [-0.26 0.13] |
|  | Caudate | -0.08 [-0.25 0.09] | -0.06 [-0.22 0.09] | -0.09 [-0.31 0.12] | 0.17 [0.05 0.30]** | 0.19 [0.08 0.3]** | 0.22 [0.04 0.4]** |
|  | Putamen | -0.13 [-0.37 0.10] | -0.04 [-0.38 0.3] | -0.19 [-0.52 0.15]* | 0.23 [0.11 0.35]** | 0.21 [0.07 0.34]** | 0.25 [0.08 0.43]** |
|  | Pallidum | 0.02 [-0.14 0.17] | 0 [-0.21 0.21] | -0.03 [-0.18 0.12] | 0.34 [0.17 0.50]** | 0.35 [0.15 0.56]** | 0.39 [0.15 0.63]** |
|  | Hippocampus | -0.23 [-0.42 -0.05]** | -0.15 [-0.44 0.14] | -0.24 [-0.45 -0.03]* | -0.31 [-0.52 -0.10]** | -0.3 [-0.54 -0.06]** | -0.22 [-0.41 -0.03]** |
|  | Amygdala | -0.14 [-0.33 0.05] | 0 [-0.2 0.21] | -0.23 [-0.54 0.09] | -0.15 [-0.31 0.01] | -0.06 [-0.31 0.19] | -0.1 [-0.3 0.1] |
|  | Accumbens | -0.16 [-0.40 0.08] | -0.19 [-0.53 0.15] | -0.3 [-0.64 0.04] | -0.10 [-0.25 0.05] | -0.14 [-0.38 0.11] | -0.19 [-0.37 -0.01]* |
|  |  |  |  |  |  |  |  |

* *p* < 0.05, uncorrected | ** *q* < 0.05, corrected | † excluded Olin in cerebellum analyses | ^#^ lithium corrected

**Supplementary Table 9.** Correlations brain and IQ; across all subjects

|  |  | IQ | | Educational attainment | |
| --- | --- | --- | --- | --- | --- |
|  |  | r ± 95% CI | ICV corrected  r ± 95% CI | r ± 95% CI | ICV corrected  r ± 95% CI |
|  | *Global measures* |  |  |  |  |
|  |  |  |  |  |  |
|  | ICV | 0.15 [0.11 0.2]** | *NA* | 0.05 [-0 0.1] | *NA* |
|  | Surface area | 0.16 [0.11 0.21]** | *NA* | 0.04 [-0.01 0.08] | *NA* |
|  | Cortical thickness | 0.09 [0.04 0.14]** | *NA* | 0.03 [-0.03 0.09] | *NA* |
|  |  |  |  |  |  |
|  |  |  |  |  |  |
|  | Total brain | 0.22 [0.18 0.26]** | 0.17 [0.12 0.22]** | 0.07 [0.01 0.12]** | 0.06 [0.01 0.11]** |
|  | Cortical gray matter | 0.22 [0.17 0.27]** | 0.17 [0.11 0.22]** | 0.08 [0.02 0.14]** | 0.08 [0.03 0.13]** |
|  | Cerebral white matter | 0.18 [0.14 0.23]** | 0.1 [0.05 0.15]** | 0.04 [-0.01 0.08] | 0.02 [-0.03 0.07] |
|  | Cerebellum gray matter† | 0.15 [0.11 0.19]** | 0.1 [0.06 0.14]** | 0.08 [0.03 0.13]** | 0.07 [0.03 0.11]** |
|  | Cerebellum white matter† | 0.13 [0.09 0.16]** | 0.08 [0.04 0.12]** | 0.06 [0.01 0.11]** | 0.05 [0.01 0.09]** |
|  | Third ventricle | -0.04 [-0.09 0.02] | -0.08 [-0.13 -0.03]** | 0.01 [-0.04 0.07] | -0.01 [-0.06 0.04] |
|  | Lateral ventricles | -0.01 [-0.05 0.04] | -0.06 [-0.11 -0.02]** | -0.01 [-0.04 0.02] | -0.02 [-0.08 0.03] |
|  |  |  |  |  |  |
|  | *Subcortical volumes* |  |  |  |  |
|  |  |  |  |  |  |
|  | Thalamus | 0.13 [0.08 0.17]** | 0.07 [0.03 0.1]** | 0.03 [-0 0.07] | 0.02 [-0.02 0.05] |
|  | Caudate | 0.08 [0.04 0.11]** | 0.03 [-0 0.06] | 0.01 [-0.02 0.05] | 0 [-0.04 0.03] |
|  | Putamen | 0.06 [0.02 0.09]** | 0.01 [-0.03 0.05] | 0.01 [-0.02 0.05] | 0 [-0.03 0.04] |
|  | Pallidum | 0.08 [0.04 0.12]** | 0.03 [-0.01 0.08] | 0.03 [-0.01 0.06] | 0.02 [-0.02 0.06] |
|  | Hippocampus | 0.16 [0.12 0.21]** | 0.11 [0.07 0.15]** | 0.07 [0.02 0.12]** | 0.05 [0.01 0.1]** |
|  | Amygdala | 0.14 [0.09 0.18]** | 0.08 [0.05 0.12]** | 0.03 [-0.02 0.08] | 0.01 [-0.03 0.05] |
|  | Accumbens | 0.09 [0.06 0.12]** | 0.05 [0.02 0.08]** | 0.01 [-0.03 0.06] | 0.01 [-0.03 0.05] |
|  |  |  |  |  |  |

* *p* < 0.05, uncorrected | ** *q* < 0.05, corrected | † excluded Olin in cerebellum analyses

**Supplementary Table 10.** Correlations brain and IQ; in the bipolar (BD; left column) and schizophrenia (SZ; right column) relatives subject groups only.

|  |  | BD relatives | SZ relatives |
| --- | --- | --- | --- |
|  |  | r ± 95% CI | r ± 95% CI |
|  | *Global measures* |  |  |
|  |  |  |  |
|  | ICV | 0.17 [0.06 0.27]** | 0.21 [0.15 0.28]** |
|  | Surface area | 0.2 [0.1 0.29]** | 0.18 [0.12 0.25]** |
|  | Cortical thickness | 0.04 [-0.09 0.17] | -0.01 [-0.07 0.06] |
|  |  |  |  |
|  |  |  |  |
|  | Total brain | 0.2 [0.1 0.29]** | 0.2 [0.13 0.27]** |
|  | Cortical gray matter | 0.21 [0.12 0.31]** | 0.17 [0.1 0.23]** |
|  | Cerebral white matter | 0.14 [0.05 0.23]** | 0.18 [0.1 0.26]** |
|  | Cerebellum gray matter† | 0.15 [0.05 0.26]** | 0.19 [0.11 0.27]** |
|  | Cerebellum white matter† | 0.08 [-0.02 0.19] | 0.16 [0.09 0.24]** |
|  | Third ventricle | 0.04 [-0.07 0.14] | 0.09 [0.02 0.16]** |
|  | Lateral ventricles | 0.02 [-0.07 0.12] | 0.12 [0.04 0.2]** |
|  |  |  |  |
|  | *Subcortical volumes* |  |  |
|  |  |  |  |
|  | Thalamus | 0.17 [0.07 0.26]** | 0.09 [0.01 0.17]** |
|  | Caudate | 0.12 [0.02 0.21]** | 0.06 [0 0.13] |
|  | Putamen | 0.12 [0.02 0.22]** | 0.03 [-0.03 0.1] |
|  | Pallidum | 0.11 [0.02 0.21]** | 0.05 [-0.02 0.11] |
|  | Hippocampus | 0.15 [0.06 0.24]** | 0.07 [0 0.13] |
|  | Amygdala | 0.12 [0.03 0.22]** | 0.09 [0.01 0.18]** |
|  | Accumbens | 0.16 [0.07 0.26]** | 0.07 [0 0.13]* |
|  |  |  |  |

* *p* < 0.05, uncorrected | ** *q* < 0.05, corrected | † excluded Olin in cerebellum analyses
